# Identifying molecular signatures of hypoxia adaptation from sex chromosomes: A case for Tibetan Mastiff based on analyses of X chromosome

**DOI:** 10.1038/srep35004

**Published:** 2016-10-07

**Authors:** Hong Wu, Yan-Hu Liu, Guo-Dong Wang, Chun-Tao Yang, Newton O. Otecko, Fei Liu, Shi-Fang Wu, Lu Wang, Li Yu, Ya-Ping Zhang

**Affiliations:** 1 State Key Laboratory for Conservation and Utilization of Bio-Resources in Yunnan, Yunnan University, Kunming, China; 2 State Key Laboratory of Genetic Resources and Evolution, and Yunnan Laboratory of Molecular Biology of Domestic Animals, Kunming Institute of Zoology, Chinese Academy of Sciences, Kunming, China; 3 Kunming College of Life Science, University of Chinese Academy of Sciences, Kunming, China; 4 Key Laboratory for Animal Genetic Diversity and Evolution of High Education in Yunnan Province, School of Life Sciences, Yunnan University, Kunming, China

## Abstract

Genome-wide studies on high-altitude adaptation have received increased attention as a classical case of organismal evolution under extreme environment. However, the current genetic understanding of high-altitude adaptation emanated mainly from autosomal analyses. Only a few earlier genomic studies paid attention to the allosome. In this study, we performed an intensive scan of the X chromosome of public genomic data generated from Tibetan Mastiff (TM) and five other dog populations for indications of high-altitude adaptation. We identified five genes showing signatures of selection on the X chromosome. Notable among these genes was angiomotin *(AMOT*), which is related to the process of angiogenesis. We sampled additional 11 dog populations (175 individuals in total) at continuous altitudes in China from 300 to 4,000 meters to validate and test the association between the haplotype frequency of *AMOT* gene and altitude adaptation. The results suggest that *AMOT* gene may be a notable candidate gene for the adaptation of TM to high-altitude hypoxic conditions. Our study shows that X chromosome deserves consideration in future studies of adaptive evolution.

Adaptation to high-altitude environments, characterised by extreme conditions like hypoxia, low temperature, and high ultraviolet radiation, has been of great interest in evolutionary biology research^1^,^2^,^3^,^4^,^5^,^6^. Numerous candidate genes which may be contributing to high-altitude adaptation, for instance those related to hypoxia (e.g., *EPAS1*, *EGLN1*, *PPARA* and *HBB* identified from human populations), energy metabolism (e.g., *PKLR*, *ENO3* and *DNAH9* identified from non-human highland animals) and DNA repair (e.g., *BCL3*, *ERCC4* and *ERCC6* identified from non-human highland animals), have been identified from studies of native highlanders with different time depths of exposure to high-altitude. These included human populations (e.g., Tibetans, Andeans and Ethiopians)^1^,^3^,^5^,^6^,^7^,^8^,^9^,^10^, wild animals (e.g., Tibetan antelopes, Tibetan wild boars, Andean waterfowls, Gray wolves, ground tits and the snub-nosed monkeys)^2^,^11^,^12^,^13^,^14^,^15^,^16^ and domesticated animals (e.g., Tibetan Mastiffs, Tibetan chickens and yaks)^4^,^17^,^18^,^19^,^20^.

Notably, most of the current understanding of genetic basis of high-altitude adaptation is based on selective scans of autosomes, despite the chromosomes of diploid organisms usually harbouring both autosomes and allosomes. Relative to autosomes, X chromosome shows reduced population size and hemizygote state in male individuals. These characters can make it be more sensitive to demographic history and selective effects^21^,^22^. Selective scans on X chromosome require a rigorous analysis design to exclude any noise caused by demographic factors from real selective signals. To date, only a few genomic studies have analysed the allosomes of highland organisms^8^. No detailed and systematic studies of allosomes in relation to high-altitude adaptation have been reported. A recent genome-wide study of *Drosophila melanogaster* populations revealed that some X-linked genes had undergone positive selection and were responsible for hypoxia tolerance^23^. Therefore, a detailed study of X chromosome, with respect to the genetic mechanism underlying the adaptation to high-altitude, is of great significance. This knowledge will boost the understanding of the complex biological feature of high-altitude adaptation.

Recently, the high-altitude adaptation of Tibetan Mastiff (TM), an ancient dog breed that migrated to the Tibetan Plateau (typically 4,500 m) with humans, has received wide attention^4^,^17^,^18^,^24^. Assessment of autosomal single nucleotide polymorphisms (SNPs) identified candidate genes, whose associated functions signified adaptation to high-altitude hypoxia. Here, we performed an X-chromosome-wide scan to investigate the adaptive strategies of the TM. By reanalysing publicly available genomic data from a TM population (10 individuals) and five other dog populations (50 individuals) from lower altitudes^17^, we identified five genes showing signatures of selection (*ALG13*, *AMOT*, *DCX*, *LHFPL1* and *TRPC5*) on the X chromosome of TM population. Notable among these genes is angiomotin *(AMOT*) gene, which was indicated to regulate the process of angiogenesis^25^,^26^,^27^,^28^. We then sampled additional 11 indigenous Chinese dog populations (175 individuals in total) from continuous altitudes ranging from 300 to 4,000 m to validate and test the association between the haplotype frequency of *AMOT* gene and altitude adaptation. The results corroborated the initial findings that *AMOT* gene may be an important target for TM’s adaptation to hypoxic conditions at their high-altitude habitat.

## Results

### Summary of mapping and SNPs calling

In the present study, the publicly available genome-wide re-sequencing data sets from six populations of 60 dogs, including a Tibetan Mastiff (TM) population (10 individuals; 4380 m altitudes) and five other dog populations (Diqing indigenous dogs, DQ; Lijiang indigenous dogs, LJ; Kunming dogs, KM; German Shepherds, GS and Yingjiang indigenous dogs, YJ; 50 individuals in total from 800–3300 m altitudes), were used^17^. Of the six dog populations, KM and GS are working dogs and the other four are indigenous dogs in China (Supplementary Tables S1 and S2).

The average raw reads of each individual from the six populations was 383, 754, 395 and the average mapping ratio was 99.73%. After filtering out duplicated reads and low-quality bases, an average of 33.96G mapped bases (~14.19 and ~11.38 folds coverage for autosomes and X chromosome respectively) for each dog were obtained (Supplementary Table S3). After SNP calling and quality control, we obtained 435, 757 high-quality SNPs from X chromosome as well as about 14.3 M from autosomes for the six populations. Compared to autosomes, X chromosome had lower SNP density (112 in X chromosome *vs*. 207 in autosomes per 50 kb) and transition/transversion (Ts/Tv) ratio (1.76 in X chromosome *vs*. 2.15 in autosomes) (Supplementary Fig. S1; Supplementary Tables S4 and S5). Based on annotations, most of the variants on X chromosome were located in the intergenic regions (about 76%), and only about 1.4% in exon regions (Supplementary Table S6). In subsequent selective scans, only the variants (435, 757 SNPs) from X chromosome were used.

### Genetic backgrounds of the six dog populations

**Figure 1:**
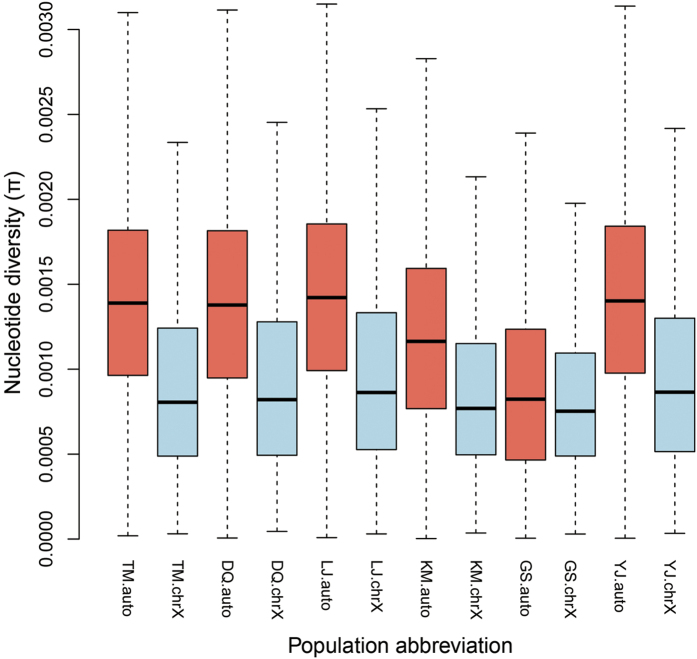
Boxplot of pairwise nucleotide diversity (π) for the six dog populations from Gou *et al*.^17^. Genetic diversity surveys were carried out on each population with non-overlapping 100 k windows across the whole X chromosome. The divergence level of nucleotide diversity on X chromosome between four dog populations and two working breeds was assessed using T-test.

The genetic diversity or pairwise nucleotide diversity^29^ (π) assessment showed that autosomes have higher levels of genetic diversity relative to X chromosome across all the six dog populations (1.0E-3 in X chromosome *vs*. 1.3E-3 in autosomes) (Fig. 1). Among these populations, the two working dog populations, i.e., KM and GS, were excluded from further selective analyses for four main reasons. First, KM and GS populations showed the lowest genetic diversity values in both autosomes and X chromosome compared to other populations, implying likely serious genetic bottlenecks faced by the two modern breeds. This finding is similar to that reported from autosomes analyses^17^. Second, principle components analysis (PCA) results based on X chromosome showed that the six populations separated into two major groups, one group included KM and GS, which are modern dog breeds, and the other group included TM, DQ, LJ and YJ, which are ancient breed and indigenous dogs in China. Therefore, the population structure estimations of X chromosome indicated that KM and GS have significantly different genetic backgrounds relative to the other four dog populations (Supplementary Fig. S2). Third, we performed phylogenetic analyses of the autosomes data sets and observed an evident split separating East Asian and European dogs (Supplementary Fig. S3). Interestingly, this kind of divergence was also reported by Frantz *et al*.^30^. In our present tree, KM and GS populations clustered together with the Western Eurasian core group. In contrast with the two modern breeds, the four dog populations (TM, DQ, LJ and YJ) showed closer relationships with East Asian core group. Fourth, from our Fst assessment of all the autosomal SNPs among different paired population comparisons (GS *vs*. YJ, GS *vs*.TM, KM *vs*. YJ, KM *vs*. TM and TM *vs*. YJ), we found that the introduction of GS or KM both resulted in a significantly higher Fst level as compared to TM *vs*. YJ pair (P < 2.2E-16) (Supplementary Fig. S4). These results may reflect the significant genetic difference between Chinese and European dogs, indicating that dogs of GS and KM belong to European lineage while dogs of TM and YJ belong to East Asia Linage. Taking all evidence together, as Tibetan Mastiff is a Chinese aboriginal breed, to avoid bias from the divergent genetic backgrounds of Chinese and European dogs, KM and GS populations were excluded from further selective analyses.

### Selective footprints of X chromosome in Tibetan Mastiff

**Figure 2:**
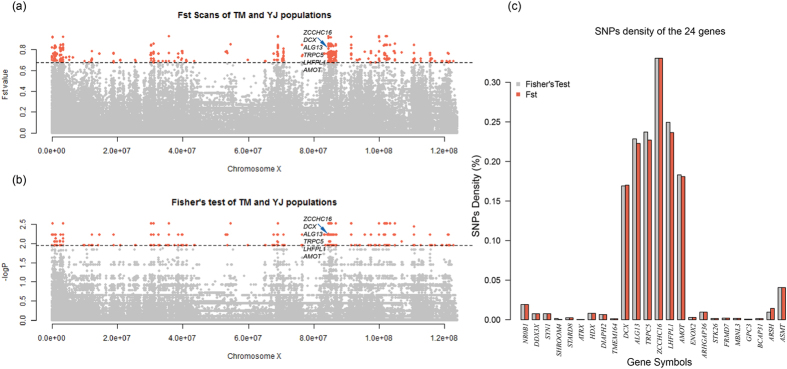
Selection scans on X chromosome for TM and YJ populations. (**a**) Fst test on X chromosome. The cutoff was set at 0.67 (1% top level). Points representing outlier values are coloured red; (**b**). Fisher’s exact test on X chromosome. The cutoff was set at 1.96 (P ≈ 0.01, after FDR corrections). Points with outlier values are coloured red. (**c**) Bar graph shows SNP densities of 24 overlapping genes between the Fst and Fisher’s tests.

The X-chromosome-wide selection scans were conducted on TM with the highest altitude of 4,380 m and on YJ population with the lowest altitude of 800 m. Based on a total of 306,678 SNPs from the two populations, using the threshold of 1% top level (equivalent to 0.68 and 1.96 after FDR correction for Fst test and Fisher’s exact test respectively), we identified 3869 (~1.2% of total SNPs) and 4049 (~1.3% of total SNPs) SNPs as outliers (Fig. 2a,b). According to the dog genome annotation (Canfam3.1)^31^, 34 and 36 genes were identified from the two respective SNPs sets. An overlap was observed between the two sets for a total of 32 genes. Moreover, 24 of these genes were characterised as protein-coding genes (Supplementary Table S7). We calculated the densities of outlier SNPs located in the 24 genes and found that six genes (*ALG13*, *AMOT*, *DCX*, *LHFPL1*, *TRPC5* and *ZCCHC16*) showed highest densities (Fig. 2c). Furthermore, the six genes were located continuously on X chromosome, and occupied a large region (~1.4 M bp). *ALG13*, *DCX*, *TRPC5* and *ZCCHC16* have functions in brain or neuron developments and cognitive competence^32^,^33^,^34^,^35^, whereas the functions of *LHFPL1* are presently unknown. Interestingly, *AMOT* (~47 kb) has been reported to code for angiomotin protein, which is located on the surface of vascular endothelial cells (VECs), and has been thought to be crucial for angiogenesis^25^,^26^,^27^,^28^.

Since both TM and DQ are high-altitude populations, 4,380 m and 3,300 m respectively, we combined the two populations and compared them against YJ, a low-altitude group, and rescanned the X chromosome following the process and criteria stated above. Interestingly, we identified 64 genes with outlier SNPs (Supplementary Table S8). Moreover, 21 of the 64 genes had featured in our previous scans (P < 1.00E-5). Except for *ZCCHC16*, the other five genes *ALG13*, *AMOT*, *DCX*, *LHFPL1*, and *TRPC5* with the highest outlier SNPs densities in the previous analyses were also observed among the 21 shared genes. Therefore, we considered the five genes as candidate positively selected genes (PSGs).

It has been reported that angiogenesis could be activated by hypoxia-inducible factor (HIF) pathway in hypoxic conditions^36^. Additionally, some genes involved in angiogenesis have been identified as targets of natural selections in high-altitude animals^11^,^13^,^19^. Thus, following the consistent featuring of *AMOT* in our analyses and its cited importance in angiogenesis, we sought to attentively assess *AMOT* with respect to high-altitude adaptation. From annotations of outlier SNPs in *AMOT*, we observed two nonsynonymous mutations (p.Ser971Ala and p.Leu1025Pro) and two synonymous mutations. Interestingly, for the two nonsynonymous mutations, the reference alleles showed significant divergent distributions between TM and YJ (86.7% in TM *vs*. 13.3% in YJ, P = 4.03E-6).

### Genetic diversity and haplotype analyses around *AMOT*

**Figure 3:**
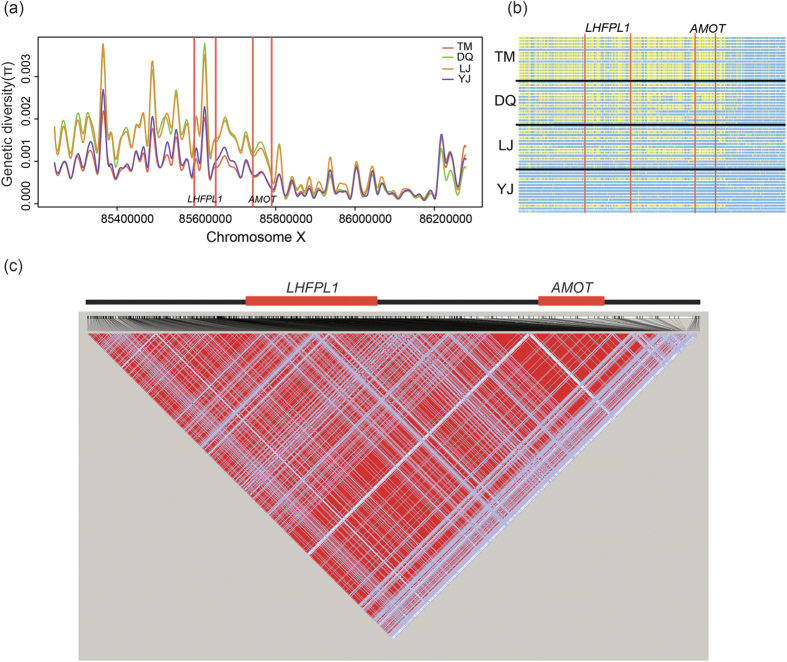
Genetic diversity surveys and haplotype analyses of the regions around *AMOT*. (**a**) Genetic diversity survey on about 1 million bp regions around *AMOT* for the four dog populations. Candidate gene regions are marked by red vertical lines. Different populations are represented with different colours. (**b**) Haplotype clusters analyses of ~500 kb around *AMOT* for the four dog populations. Size and locations of *LHFPL1* and *AMOT* are marked by red vertical lines. For each polymorphic locus of a chromosome or haplotype, point with yellow colour represents high-altitude allele, and that with light blue colour represents low-altitude allele. (**c**) Linkage disequilibrium (LD) analysis on TM population from the same region which is used for haplotype cluster analyses.

The genetic diversity (π) scans on about 1 million bp region, covering the *AMOT* gene for four dog populations (TM, DQ, LJ and YJ), were calculated. TM and YJ had the lowest genetic diversity compared to DQ and LJ populations (Fig. 3a). This pattern may imply a gradual change of allele frequency among the four populations. A further haplotype analysis based on a region about 500 kb covering *AMOT* showed that TM and YJ populations exhibited obvious divergence in haplotypes, and a gradual change from YJ to TM (Fig. 3b), which is consistent with the results from the initial genetic diversity analysis. Moreover, in the same region, LD analysis showed that TM population had a strong LD block across the whole region and the strongest tendency located in *AMOT* (Fig. 3c; Supplementary Fig. S5).

Given that genetic drift can also generate similar genetic patterns as positive selection did, we performed simulations for 1,000,000 times to generate allele frequency data sets of TM and YJ populations with *ms* tools^37^ for the *AMOT* region (sequence length 47,544 bp, including 108 SNPs). Then we used these data to test whether the observed TM-YJ genetic divergence could be due to genetic drift instead of selection. For each run, we calculated Fst values of the 108 SNPs from the simulated data sets, and compared the result to the Fst values from the real data. Our analyses showed that the probability of the real Fst values attributable to genetic drift was only 2.32E-3, implying that the genetic pattern observed on *AMOT* was likely not caused by genetic drift, and *AMOT* may be a target of positive selection during adaptation to hypoxia.

### Correlation between *AMOT* haplotype frequency and altitude

**Figure 4:**
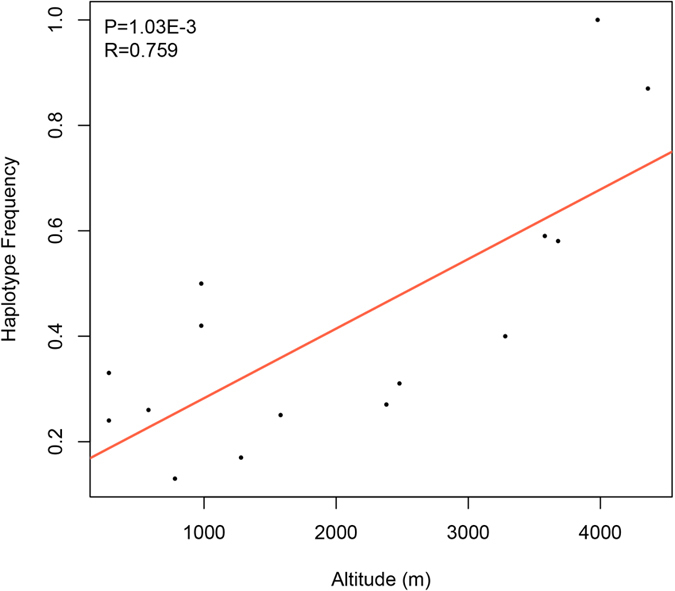
Correlation between haplotype frequency and altitude among 15 dog populations. Two fragments around *AMOT* were genotyped and included 11 SNPs. Frequency of the TM major haplotype (‘CGTTCGCTTTG’) has been calibrated with gender information.

Inspired by the observed gradual variation in genetic diversity and haplotype distributions among the four dog populations, we additionally sampled 175 individuals of 11 aboriginal dog populations from different areas in China (altitude range: 300 m to 4,000 m) to further validate the selective signatures of *AMOT* (Supplementary Tables S9 and S10; Supplementary Fig. S6). In this part, we genotyped two fragments around *AMOT* to test the correlation between haplotype frequency and altitude. The two fragments were about 112 kb apart and included 11 SNPs. Combining the four dog populations used in X-chromosome-wide selective analyses, we built haplotypes of the 11 SNPs from all the 15 dog populations (215 individuals in total). Interestingly, one kind of haplotype (‘CGTTCGCTTTG’) occupied a very high ratio (about 82% on average) in Tibetan Mastiff populations (Table 1). Moreover, there was a significant correlation between the TM’s major haplotype frequency and altitude (P = 1.03E-3) (Fig. 4).

Taken together, the scans of X chromosome of TM and other dog populations, and the subsequent analyses of the genetic diversity, haplotype distributions, as well as the correlation between haplotype frequency and altitude, all provide evidence suggesting that *AMOT* could be a key target gene of positive selections, and could be associated with high-altitude hypoxia tolerance among Tibetan Mastiff.

## Discussion

Several selective scans on X chromosome have been reported in previous animal domestications and breeding studies. Specific regions or genes have shown likely involvement in the process of domestications of pig and sheep^38^,^39^,^40^. However, the present study is the first to perform detailed X-chromosome-wide scans to address the genetic basis of high-altitude adaptation, a notable contribution in filling up the knowledge gaps in the molecular mechanisms of high-altitude adaptation. In addition, relative to previous analyses of X chromosome in animal domestications studies, in which the genome-wide data were mainly generated from microarray or pooling re-sequencing platforms, our present work has used more high-quality data sets with independent re-sequenced libraries and good sequencing coverage, increasing the possibility of identifying high-quality SNPs and more signatures of positive selection (Supplementary Tables S3 and S5).

Here, we chose Tibetan Mastiff, which is a typical representative of domesticated animal that migrated to the high-altitude plateau with humans, as our study model. TM has been investigated in several studies for its high-altitude adaptation via autosome genome analyses^4^,^17^,^18^. From these analyses, two PSGs, i.e., *EPAS1* and *HBB*, which are associated with HIF pathway and oxygen transportation, and one PSG, i.e., *PLXNA4*, which functions in angiogenesis by interacting with *VEGF*^41^, have been reported as targeted candidate genes for adaptation to high-altitude^18^. In our current selective scans on X chromosome in TM and other dog populations from different elevation gradients (from 800 to 4380m), we have identified five new PSGs. Among these PSGs, *AMOT* gene has been reported to act in the process of angiogenesis^25^,^26^,^27^,^28^. With simulation analysis and genotyping experiments on extra 11 dog populations, we propose that this gene may have undergone selective effects during the adaptation to hypoxic conditions in TM. The genetic diversity levels of *AMOT* in TM and YJ populations based on a large genetic region were similar low. As high and low elevations represent two different environments, divergent selective pressures may have acted on *AMOT* in TM and YJ resulting in the similar genetic diversity patterns, although a narrowed scan showed a marked haplotype divergence between the two populations. Interestingly, studies on Atlantic Salmons and dog populations have observed signals of divergent selections in some regions, in which the selective effects caused increased genetic differentiation between populations and reduced genetic diversity at linked loci^42^,^43^.

Although several angiogenesis-related genes, including *VEGF*, *VAV3*, *NOS3*, *SRF*, *TXNRD2*, *ADAM17* and *RNASE4*, have been proposed as candidate genes in the adaptation of highland human and wild animals to high-altitude conditions^8^,^10^,^11^,^13^,^16^,^19^, our study for the first time identified that *AMOT*, which is also an angiogenesis related gene, may have played a key role in the high-altitude adaptation of TM. Hence, it is likely that, besides the HIF pathway, angiogenesis also played a similarly critical role in the adaptation of TM populations to their high-altitude hypoxic habitat.

This study presents important insights for understanding the contribution of sex chromosomes to hypoxia adaptation, as most previous genome-wide studies have often excluded sex chromosome because it represents a different effective population size. Relative to autosomes, our genetic diversity assessments demonstrated that X chromosome had lower genetic diversity across all six dog populations (Fig. 1), consistent with the findings of a recent genome-wide study among human populations^22^. Although population structures estimated from X chromosomal SNPs showed similar patterns with Gou *et al*.^17^, PCA based on allosomes could not clearly distinguish each dog population. On the other hand, the Fst distributions of autosomes and X chromosome between YJ and TM showed that allosome had greater genetic divergence compared to autosomes (P = 2.2E-16) (Supplementary Fig. S7). Taken together, these findings lead to the postulation that X chromosome might have suffered stronger selection pressure or genetic drift effects than autosomes, and that allosome might be more sensitive to selection pressure, resulting in a different population structure.

In summary, our study suggests that X chromosome, like autosomes, may have a contribution in the adaptation of Tibetan Mastiff to hypoxic conditions at high-altitudes, indicating that X chromosome warrants a more attention in future studies of adaptive evolution. In addition, the identification of *AMOT* as a target candidate gene provides a foundation for future functional studies to further elucidate its role in adaptation to hypoxia.

## Methods

All methods were performed in accordance with the guidelines approved by the Kunming Institute of Zoology, Chinese Academy of Sciences. All experimental protocols were approved by the Kunming Institute of Zoology, Chinese Academy of Sciences.

### Reads mapping and SNPs calling

Raw sequence reads were mapped to the dog reference genome (version: Canfam3.1) using BWA-MEM (version: 0.7.8-r455)^44^, and Bam files generated by SAMtools (version: 0.1.18)^45^. PICARD software packages (version: 1.87; http://picard.sourceforge.net) was used to remove duplicate sequences in each individual, then all the Bam files were processed with a standard pipeline for local re-alignment and base-recalibration in the Genome Analysis Tool Kits (GATK) (version: 2.5-2-gf57256b)^46^.

Genome-wide SNPs calling were handled by UnifiedGenotypeCaller module in GATK. Raw SNPs were recalibrated with the Variant Quality Score Recalibration (VQSR) module, where a list of known or verified SNPs/INDELs was downloaded from Ensembl Database and used as the training data set. Then the recalibrated SNPs were filtered with rules as described below: 1) SNPs that were within 5 base pairs proximity to inserts and deletions (INDELs) were filtered out, and the INDELs also abandoned; 2) SNP locus at which the ratio of individuals missing SNP detection was greater than 20% of all the samples was excluded; 3) Only bi-allelic SNPs were retained; 4) The remaining SNPs were sorted by their depths, with depths ranging from 2.5% to 97.5% retained; and 5) SNPs with Quality value greater than 40 were retained. Finally, all high-quality SNPs were annotated by ANNOVAR^47^.

### Genetic background survey of the six populations

With the availability of complete gender information for the six dog populations, we made corrections to the allele frequencies of the study populations before assessing population genetic diversity and structure. Here, we first computed pairwise nucleotide diversity (π)^29^ of autosomes and X chromosome among the six dog populations using custom Perl scripts (non-overlapped 100 k window-size), then principal component analysis (PCA) and population structure estimations were conducted with EIGENSOFT (version: 6.0.1)^48^ and ADMIXTURE (version: 1.23)^49^. Before population structure estimations, some SNPs were filtered out, in a process referred to as SNPs thinning, using PLINK (version: v1.07; http://pngu.mgh.harvard.edu/purcell/plink/; Parameter: –indep-pairwise 50 10 0.1), to make sure that each SNP evolved independently. Based on the whole autosomal SNPs from our downloaded genome-wide data sets^17^, and the recently published genome-wide data sets of dogs and wolves^50^, we employed SNPhylo^51^ to construct the phylogenetic relationships of the six dog populations. In addition, Fst surveys on the five population pairs (KM *vs*. YJ, KM *vs*. TM, GS *vs*. YJ, GS *vs*. TM, and YJ *vs*. TM) were also conducted.

### Selection scans on X chromosome

Two approaches were applied for positive selection scans: Fst test, which reflects the genetic differences among populations^52^. The other is Fisher’s exact test, which does not need biological hypothesis and can estimate the probability of divergence in allele frequency among populations. Only the top 1% of SNPs with high Fst values in Fst test, or small *P*-values (after FDR correction) in Fisher’s exact test, were considered as candidate loci of selection.

### Genetic diversity and haplotype surveys in candidate genes

The genetic diversity (π) scans of regions around candidate genes were performed with our custom Perl scripts, applying 10 k-bp non-steps windows. Prior to our haplotype analyses, haplotypes of the four dog populations (TM, DQ, LJ and YJ) were inferred by SHAPEIT software^53^. To increase the haplotypes estimation accuracy, the genetic map of domestic dogs downloaded from a recent work of Auton *et al*.^54^ was used in the haplotype construction. Linkage disequilibrium (LD) and haplotype clusters of regions around candidate genes were then analysed using Haploview (version: 4.2)^55^ and R (version: 3.1.0) software respectively.

### Simulation tests of Candidate genes

We applied dense computational simulations to test whether the selective signals of *AMOT* gene were caused by genetic drift instead of selection. First, using G-PhoCS tools^56^, 1463 neutral loci of X chromosome from ten female individuals of the TM and YJ populations were chosen for demographic history inference. It has been reported that mutation rate is 1.3 times higher in the autosome than in the X chromosome^57^, and that the mutation rate in the autosome of dogs is 2.2 × 10^−9^ per year^58^. Therefore, for our simulation, we used 1.692 × 10^−9^ per year as the mutation rate in the X chromosome. Our results showed that TM diverged from YJ about 3,400 years ago, and the effective population size of their common ancestor was approximately 37,800. On the other hand, the effective population size of extant YJ and TM was 8,681 and 4,058 respectively. With parameters drawn from previous analyses, we used *ms* tools^37^ to simulate the allele frequency of 108 SNPs spanning the whole *AMOT* region (about 47 k bps) for both TM and YJ populations in a neutral model. In each simulation, we calculated the Fst values of simulated data sets, and compared them to those computed from the real data sets, with the significance of the divergence measured by t-test. After 1,000,000 simulations, the distribution of all P-values was evaluated to determine whether selective footprints that were produced by the real data sets were generated by genetic drift.

### Genotyping and *AMOT* haplotype correlation tests

To verify the selective signals of *AMOT* identified from X-chromosome-wide scans, we sampled additional 175 dogs from 11 different places in China (altitude: 300 m to 4000 m) and added them into our present study (Supplementary Tables S9 and S10; Supplementary Fig. S6). Total genomic DNA was extracted from peripheral venous blood using the Phenol/Chloroform method. Polymerase Chain Reaction (PCR) was performed using ExTaq (TaKaRa; Dalian, China). We genotyped two fragments around *AMOT*. To genotype the first fragment, which is located in the sixth intron of *AMOT*, the following primers, F1: 5′-CACCATACCCAATCTCTGTCTA-3′and R1: 5′-GAAGGTATGAAGGTGCCTAGGA-3′, and PCR conditions, 94 °C for 30 sec, 57 °C for 30 sec, 72 °C for 1 min, and 32 cycles, were used. For the second fragment, a flanking sequence upstream of *AMOT*, primer pair of F2: 5′-AAGTCAGAGCAATGGGAAGC-3′ and R2: 5′-GTTTGGTGGGCTGTGAAGAC-3′, as well as PCR conditions of 94 °C for 30 sec, 65 °C for 30 sec, 72 °C for 1 min, and 32 cycles were used. After quality control procedures, we obtained 350 sequences with high-quality. The newly determined sequences have been deposited in GenBank: KX245540-KX245889. We then joined the two fragments into one continuous sequence (containing 11 SNPs) for each individual, and used PHASE (version: 2.1)^59^,^60^ to build haplotypes of the all SNPs for the overall dataset of 15 dog populations. Based on the gender information for all samples, we corrected the frequency of the TM major haplotype in each dog population. Finally, the correlation analysis between haplotype frequency and altitude was assessed via R (version: 3.1.0).

## Additional Information

**Accession codes**: All newly generated sequences of the 11 dog populations have been deposited in GenBank (KX245540-KX245889).

**How to cite this article**: Wu, H. *et al*. Identifying molecular signatures of hypoxia adaptation from sex chromosomes: A case for Tibetan Mastiff based on analyses of X chromosome. *Sci. Rep*. **6**, 35004; doi: 10.1038/srep35004 (2016).

## Supplementary Material

Supplementary Information

## Figures and Tables

**Table 1:** Frequencies of the TM major haplotype among all the 15 dog populations.

Population	Altitude (m)	Number of Chromosomes	Number of the TM major haplotype*	Frequency of the TM major haplotype
HEB	300	27	9	0.33
PZ	300	21	5	0.24
YA	600	27	7	0.26
YJ	800	15	2	0.13
XA	1000	26	13	0.50
CD	1000	36	15	0.42
SM	1300	30	5	0.17
YX	1600	16	4	0.25
LJ1	2400	15	4	0.27
LJ2	2500	16	5	0.31
DQ	3300	15	6	0.40
HY	3700	19	11	0.58
MQ	3600	17	10	0.59
YS	4000	27	27	1.00
TM	4380	15	13	0.87

^*^The sequence of the TM major haplotype is ‘**C**GT**TC**G**C**TT**T**G’, and characters with underlines represent the non-reference alleles.

## References

[b1] Beall C. M. . Natural selection on EPAS1 (HIF2 alpha) associated with low hemoglobin concentration in Tibetan highlanders. Proc. Natl. Acad. Sci. USA 107, 11459–11464 (2010). 20534544 10.1073/pnas.1002443107 PMC2895075

[b2] Natarajan C. . Convergent Evolution of Hemoglobin Function in High-Altitude Andean Waterfowl Involves Limited Parallelism at the Molecular Sequence Level. PLoS Genet. 11, e1005681 (2015). 26637114 10.1371/journal.pgen.1005681 PMC4670201

[b3] Simonson T. S. . Genetic Evidence for High-Altitude Adaptation in Tibet. Science 329, 72–75 (2010). 20466884 10.1126/science.1189406

[b4] Wang G. D. . Genetic Convergence in the Adaptation of Dogs and Humans to the High-Altitude Environment of the Tibetan Plateau. Genome Biol. Evol. 6, 2122–2128 (2014). 25091388 10.1093/gbe/evu162 PMC4231634

[b5] Xu S. H. . A Genome-Wide Search for Signals of High-Altitude Adaptation in Tibetans. Mol. Biol. Evol. 28, 1003–1011 (2011). 20961960 10.1093/molbev/msq277

[b6] Yi X. . Sequencing of 50 Human Exomes Reveals Adaptation to High Altitude. Science 329, 75–78 (2010). 20595611 10.1126/science.1190371 PMC3711608

[b7] Alkorta-Aranburu G. . The Genetic Architecture of Adaptations to High Altitude in Ethiopia. Plos Genet. 8, e1003110 (2012). 23236293 10.1371/journal.pgen.1003110 PMC3516565

[b8] Bigham A. . Identifying Signatures of Natural Selection in Tibetan and Andean Populations Using Dense Genome Scan Data. Plos Genet. 6, e1001116 (2010). 20838600 10.1371/journal.pgen.1001116 PMC2936536

[b9] Peng Y. . Genetic Variations in Tibetan Populations and High-Altitude Adaptation at the Himalayas. Mol. Biol. Evol. 28, 1075–1081 (2011). 21030426 10.1093/molbev/msq290

[b10] Scheinfeldt L. B. . Genetic adaptation to high altitude in the Ethiopian highlands. Genome Biol. 13, R1 (2012). 22264333 10.1186/gb-2012-13-1-r1 PMC3334582

[b11] Ge R. L. . Draft genome sequence of the Tibetan antelope. Nat. Commun. 4, e1858 (2013). 10.1038/ncomms2860 PMC3674232 23673643

[b12] Li M. Z. . Genomic analyses identify distinct patterns of selection in domesticated pigs and Tibetan wild boars. Nat. Genet. 45, 1431–1438 (2013). 24162736 10.1038/ng.2811

[b13] Qu Y. . Ground tit genome reveals avian adaptation to living at high altitudes in the Tibetan plateau. Nat. Commun. 4, e2071 (2013). 10.1038/ncomms3071 23817352

[b14] Zhang W. P. . Hypoxia Adaptations in the Grey Wolf (Canis lupus chanco) from Qinghai-Tibet Plateau. Plos Genet. 10, e1004466 (2014). 25078401 10.1371/journal.pgen.1004466 PMC4117439

[b15] Galen S. C. . Contribution of a mutational hot spot to hemoglobin adaptation in high-altitude Andean house wrens. Proc. Natl. Acad. Sci. USA 112, 13958–13963 (2015). 26460028 10.1073/pnas.1507300112 PMC4653164

[b16] Yu L. . Genomic analysis of snub-nosed monkeys (*Rhinopithecus*) identifies genes and processes related to high-altitude adaptation. Nat. Genet. 48, 947–952 (2016). 27399969 10.1038/ng.3615

[b17] Gou X. . Whole-genome sequencing of six dog breeds from continuous altitudes reveals adaptation to high-altitude hypoxia. Genome Res. 24, 1308–1315 (2014). 24721644 10.1101/gr.171876.113 PMC4120084

[b18] Li Y. . Population Variation Revealed High-Altitude Adaptation of Tibetan Mastiffs. Mol. Biol. Evol. 31, 1200–1205 (2014). 24520091 10.1093/molbev/msu070

[b19] Qiu Q. . The yak genome and adaptation to life at high altitude. Nat. Genet. 44, 946–949 (2012). 22751099 10.1038/ng.2343

[b20] Wang M. S. . Genomic Analyses Reveal Potential Independent Adaptation to High Altitude in Tibetan Chickens. Mol. Biol. Evol. 32, 1880–1889 (2015). 25788450 10.1093/molbev/msv071

[b21] Meisel R. P. & Connallon T. The faster-X effect: integrating theory and data. Trends Genet. 29, 537–544 (2013). 23790324 10.1016/j.tig.2013.05.009 PMC3755111

[b22] Gottipati S., Arbiza L., Siepel A., Clark A. G. & Keinan A. Analyses of X-linked and autosomal genetic variation in population-scale whole genome sequencing. Nat. Genet. 43, 741–743 (2011). 21775991 10.1038/ng.877 PMC3145052

[b23] Zhou D. . Experimental selection of hypoxia-tolerant Drosophila melanogaster. Proc. Natl. Acad. Sci. USA 108, 2349–2354 (2011). 21262834 10.1073/pnas.1010643108 PMC3038716

[b24] Li Y. & Zhang Y. P. High genetic diversity of Tibetan Mastiffs revealed by mtDNA sequences. Chinese Sci. Bull. 57, 1483–1487 (2012).

[b25] Bratt A. . Angiomotin regulates endothelial cell-cell junctions and cell motility. J. Biol. Chem. 280, 34859–34869 (2005). 16043488 10.1074/jbc.M503915200

[b26] Ernkvist M. . The Amot/Patj/Syx signaling complex spatially controls RhoA GTPase activity in migrating endothelial cells. Blood 113, 244–253 (2009). 18824598 10.1182/blood-2008-04-153874 PMC2614636

[b27] Troyanovsky B., Levchenko T., Mansson G., Matvijenko O. & Holmgren L. Angiomotin: an angiostatin binding protein that regulates endothelial cell migration and tube formation. J. Cell Biol. 152, 1247–1254 (2001). 11257124 10.1083/jcb.152.6.1247 PMC2199208

[b28] Aase K. . Angiomotin regulates endothelial cell migration during embryonic angiogenesis. Genes Dev. 21, 2055–2068 (2007). 17699752 10.1101/gad.432007 PMC1948860

[b29] Kimura M. Genetic variability maintained in a finite population due to mutational production of neutral and nearly neutral isoalleles. Genet. Res. 11, 247–269 (1968). 5713805 10.1017/s0016672300011459

[b30] Frantz L. A. . Genomic and archaeological evidence suggest a dual origin of domestic dogs. Science 352, 1228–1231 (2016). 27257259 10.1126/science.aaf3161

[b31] Lindblad-Toh K. . Genome sequence, comparative analysis and haplotype structure of the domestic dog. Nature 438, 803–819 (2005). 16341006 10.1038/nature04338

[b32] Bissar-Tadmouri N. . X Chromosome Exome Sequencing Reveals a Novel ALG13 Mutation in a Nonsyndromic Intellectual Disability Family With Multiple Affected Male Siblings. Am. J. Med. Genet. A 164, 164–169 (2014). 10.1002/ajmg.a.36233 24501762

[b33] Gleeson J. G. . Doublecortin, a brain-specific gene mutated in human X-linked lissencephaly and double cortex syndrome, encodes a putative signaling protein. Cell 92, 63–72 (1998). 9489700 10.1016/s0092-8674(00)80899-5

[b34] Sossey-Alaoui K. . Molecular cloning and characterization of TRPC5 (HTRP5), the human homologue of a mouse brain receptor-activated capacitative Ca2+ entry channel. Genomics 60, 330–340 (1999). 10493832 10.1006/geno.1999.5924

[b35] Irie M. . Cognitive Function Related to the Sirh11/Zcchc16 Gene Acquired from an LTR Retrotransposon in Eutherians. Plos Genet. 11 (2015). 10.1371/journal.pgen.1005521 PMC4581854 26402067

[b36] Rey S. & Semenza G. L. Hypoxia-inducible factor-1-dependent mechanisms of vascularization and vascular remodelling. Cardiovasc. Res. 86, 236–242 (2010). 20164116 10.1093/cvr/cvq045 PMC2856192

[b37] Hudson R. R. Generating samples under a Wright-Fisher neutral model of genetic variation. Bioinformatics 18, 337–338 (2002). 11847089 10.1093/bioinformatics/18.2.337

[b38] Rubin C.-J. . Strong signatures of selection in the domestic pig genome. Proc. Natl. Acad. Sci. USA 109, 19529–19536 (2012). 23151514 10.1073/pnas.1217149109 PMC3511700

[b39] Ma Y., Zhang H., Zhang Q. & Ding X. Identification of Selection Footprints on the X Chromosome in Pig. Plos One 9, e94911 (2014). 24740293 10.1371/journal.pone.0094911 PMC3989256

[b40] Moradi M. H., Nejati-Javaremi A., Moradi-Shahrbabak M., Dodds K. G. & McEwan J. C. Genomic scan of selective sweeps in thin and fat tail sheep breeds for identifying of candidate regions associated with fat deposition. BMC Genet. 13, 10; 1186/1471-2156-13-10 (2012). 10.1186/1471-2156-13-10 PMC3351017 22364287

[b41] Kigel B., Rabinowicz N., Varshavsky A., Kessler O. & Neufeld G. Plexin-A4 promotes tumor progression and tumor angiogenesis by enhancement of VEGF and bFGF signaling. Blood 118, 4285–4296 (2011). 21832283 10.1182/blood-2011-03-341388

[b42] Tezuka A. . Divergent selection for opsin gene variation in guppy (Poecilia reticulata) populations of Trinidad and Tobago. Heredity (Edinb) 113, 381–389 (2014). 24690753 10.1038/hdy.2014.35 PMC4220713

[b43] Pilot M. . Diversifying Selection Between Pure-Breed and Free-Breeding Dogs Inferred from Genome-Wide SNP Analysis. G3 Bethesda 6, 2285–2298 (2016). 27233669 10.1534/g3.116.029678 PMC4978884

[b44] Li H. & Li H. Aligning sequence reads, clone sequences and assembly contigs with BWA-MEM. arXiv (1303.3997). Eprint Arxiv 1303 (2013).

[b45] Li H. . The Sequence Alignment/Map format and SAMtools. Bioinformatics 25, 2078–2079 (2009). 19505943 10.1093/bioinformatics/btp352 PMC2723002

[b46] DePristo M. A. . A framework for variation discovery and genotyping using next-generation DNA sequencing data. Nat. Genet. 43, 491–498 (2011). 21478889 10.1038/ng.806 PMC3083463

[b47] Wang K., Li M. & Hakonarson H. ANNOVAR: functional annotation of genetic variants from high-throughput sequencing data. Nucleic Acids Res. 38, e164 (2010). 20601685 10.1093/nar/gkq603 PMC2938201

[b48] Patterson N., Price A. L. & Reich D. Population structure and eigenanalysis. Plos Genet. 2, 2074–2093 (2006). 10.1371/journal.pgen.0020190 PMC1713260 17194218

[b49] Alexander D. H., Novembre J. & Lange K. Fast model-based estimation of ancestry in unrelated individuals. Genome Res. 19, 1655–1664 (2009). 19648217 10.1101/gr.094052.109 PMC2752134

[b50] Wang G. D. . Out of southern East Asia: the natural history of domestic dogs across the world. Cell Res. 26, 21–33 (2016). 26667385 10.1038/cr.2015.147 PMC4816135

[b51] Lee T. H., Guo H., Wang X., Kim C. & Paterson A. H. SNPhylo: a pipeline to construct a phylogenetic tree from huge SNP data. BMC Genomics 15, 162 (2014). 24571581 10.1186/1471-2164-15-162 PMC3945939

[b52] Akey J. M., Zhang G., Zhang K., Jin L. & Shriver M. D. Interrogating a high-density SNP map for signatures of natural selection. Genome Res. 12, 1805–1814 (2002). 12466284 10.1101/gr.631202 PMC187574

[b53] Delaneau O., Marchini J. & Zagury J. F. A linear complexity phasing method for thousands of genomes. Nat. Methods 9, 179–181 (2012). 10.1038/nmeth.1785 22138821

[b54] Auton A. . Genetic Recombination Is Targeted towards Gene Promoter Regions in Dogs. Plos Genet. 9, e1003984 (2013). 24348265 10.1371/journal.pgen.1003984 PMC3861134

[b55] Barrett J. C., Fry B., Maller J. & Daly M. J. Haploview: analysis and visualization of LD and haplotype maps. Bioinformatics 21, 263–265 (2005). 15297300 10.1093/bioinformatics/bth457

[b56] Gronau I., Hubisz M. J., Gulko B., Danko C. G. & Siepel A. Bayesian inference of ancient human demography from individual genome sequences. Nat Genet 43, 1031–1034 (2011). 21926973 10.1038/ng.937 PMC3245873

[b57] Ebersberger I., Metzler D., Schwarz C. & Paabo S. Genomewide comparison of DNA sequences between humans and chimpanzees. Am. J. Hum. Genet. 70, 1490–1497 (2002). 11992255 10.1086/340787 PMC379137

[b58] Kumar S. & Subramanian S. Mutation rates in mammalian genomes. Proc. Natl. Acad. Sci. USA 99, 803–808 (2002). 11792858 10.1073/pnas.022629899 PMC117386

[b59] Stephens M., Smith N. J. & Donnelly P. A new statistical method for haplotype reconstruction from population data. Am. J. Hum. Genet. 68, 978–989 (2001). 11254454 10.1086/319501 PMC1275651

[b60] Stephens M. & Donnelly P. A comparison of bayesian methods for haplotype reconstruction from population genotype data. Am. J. Hum. Genet. 73, 1162–1169 (2003). 14574645 10.1086/379378 PMC1180495

