# Supplementary material for: Identifying molecular signatures of hypoxia adaptation from sex chromosomes: A case for Tibetan Mastiff based on analyses of X chromosome
Source: Sci Rep. 2016 Oct 7;6:35004. doi: 10.1038/srep35004 (PMC5054530; doi:10.1038/srep35004)
Supplement: Supplementary Information [file srep35004-s1.doc]

**Identifying molecular signatures of hypoxia adaptation from sex chromosomes: A case for Tibetan Mastiff based on analyses of X chromosome**

Hong Wu^1, 2, 3, 4+^, Yan-Hu Liu^1, 4+^, Guo-Dong Wang^2^, Chun-Tao Yang^1, 4^, Newton O. Otecko^2, 3^, Fei Liu^2, 3^, Shi-Fang Wu^2^, Lu Wang^1, 4,*^, Li Yu^1, 4*^&Ya-Ping Zhang^1, 2*^

^1^State Key Laboratory for Conservation and Utilization of Bio-Resources in Yunnan, Yunnan University, Kunming, China

^2^State Key Laboratory of Genetic Resources and Evolution, and Yunnan Laboratory of Molecular Biology of Domestic Animals, Kunming Institute of Zoology, Chinese Academy of Sciences, Kunming, China

^3^Kunming College of Life Science, University of Chinese Academy of Sciences, Kunming, China

^4^Key Laboratory for Animal Genetic Diversity and Evolution of High Education in Yunnan Province, School of Life Sciences, Yunnan University, Kunming, China

*correspondence should be addressed to L.W. (wanglu@ynu.edu.cn), L.Y. (yuli@ynu.edu.cn) or Y.P.Z. (zhangyp@mail.kiz.ac.cn)

^+^these authors contributed equally to this work


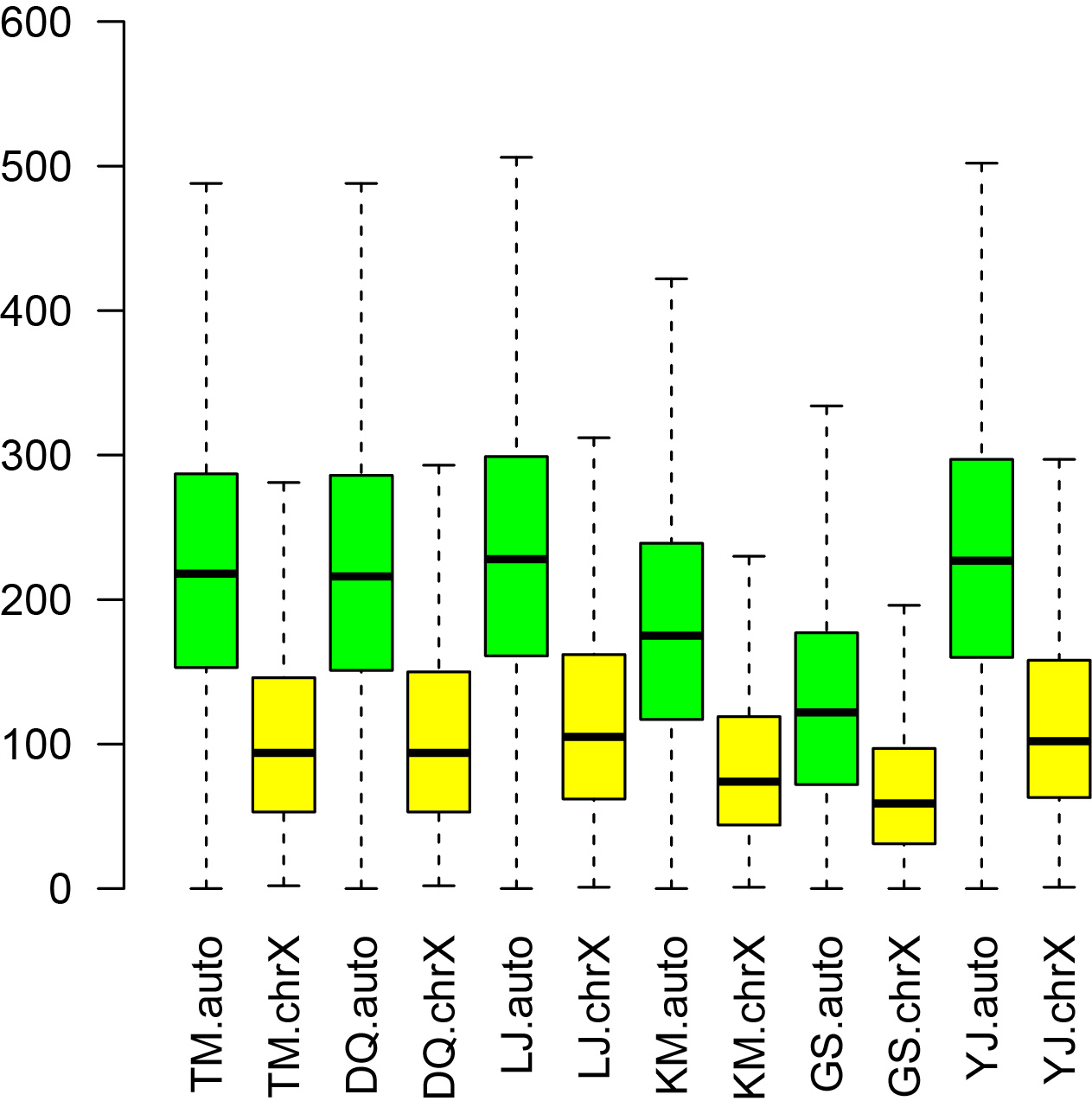


**Supplementary Fig. S1. SNP densities of autosomes and X chromosome for the six populations from Gou *et al.* (2014).** Each SNP density value was calculated in non-overlapped 50k bp windows. Boxplots with colour “green” is for autosomes and colour “yellow” is for X chromosome.


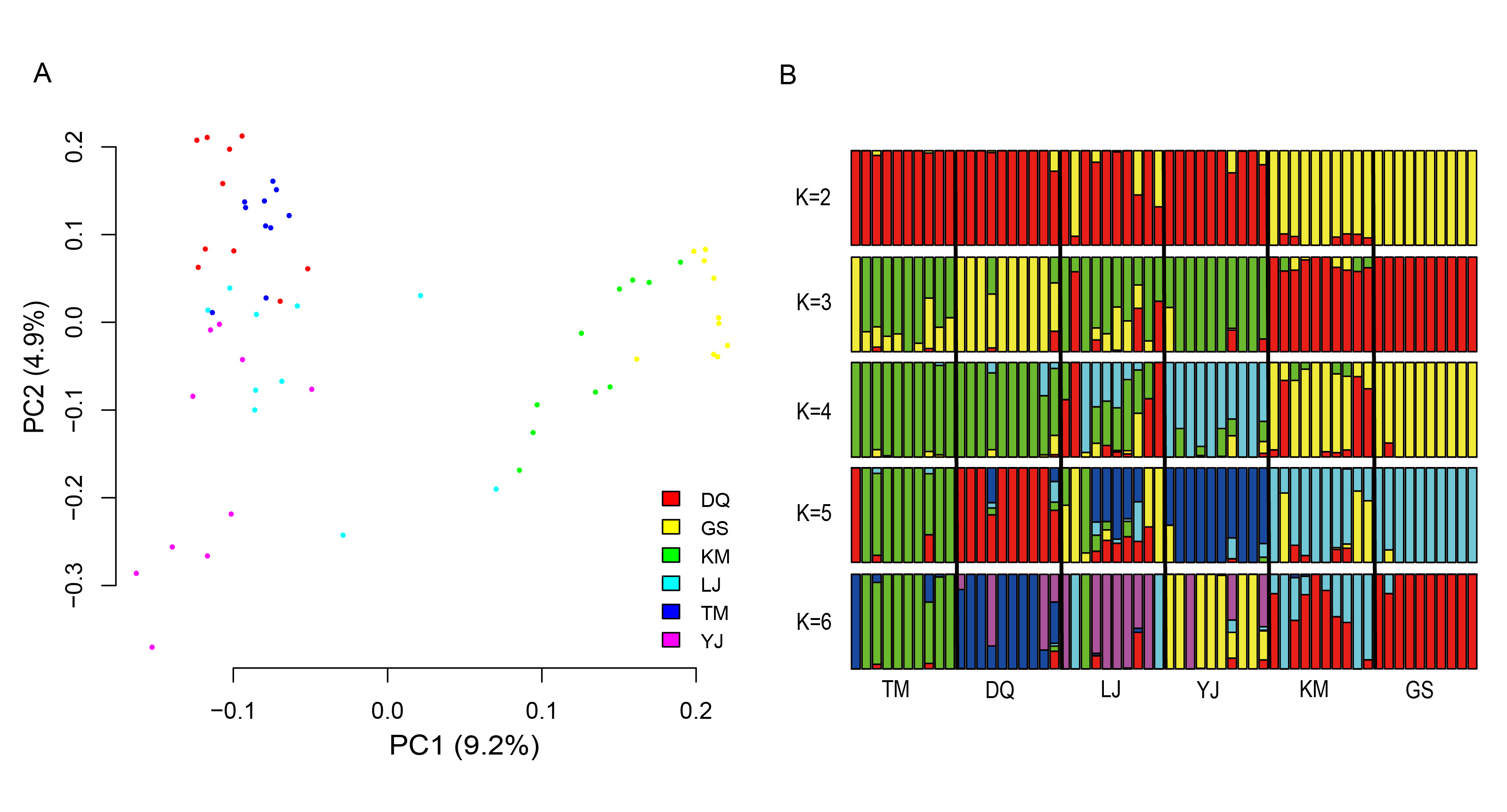


**Supplementary Fig. S2. Principal Component Analysis (PCA) and Population Structure for six populations from Gou *et al.* (2014). based on X chromosome data**. (A). PCA result. The whole SNPs (435,757) dataset of the six breeds were used, and only PC1 and PC2 were shown; (B). Population Structure result. To avoid the bias resulting from LD, all SNPs have been thinned by PLINK. K=2 presents the best estimation.


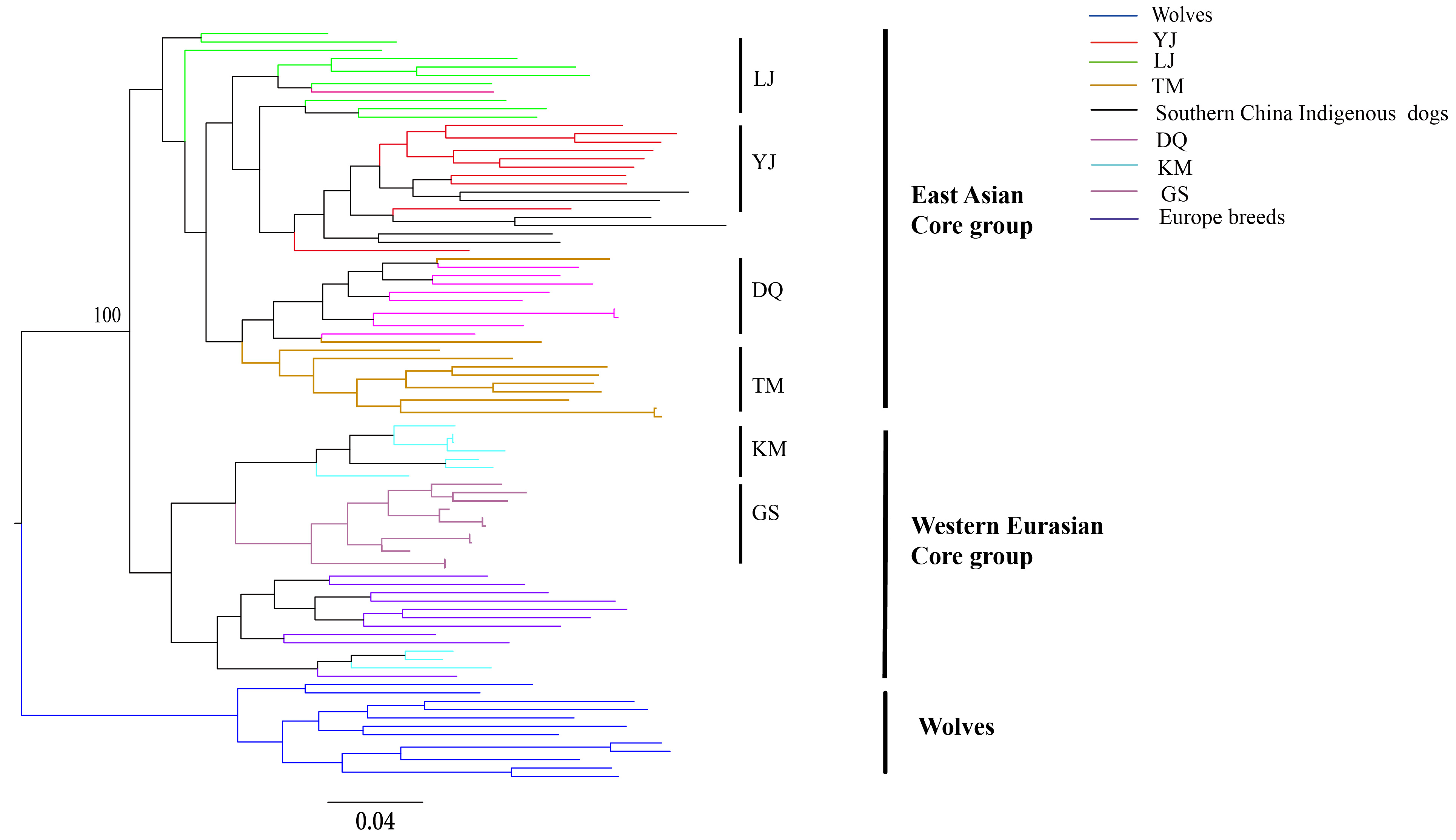


**Supplementary Fig. S3. Phylogenetic analyses of the six dog populations** **from Gou et al. (2014) and others.** The phylogenetic relationship of the dog populations, including six dog populations from Gou *et al* (2014) and those from Wang *et al.* (2016), was constructed based on the whole autosomal SNPs. GS and KM were located in Western Eurasian core group and the other four dog populations were clustered with East Asian core group.


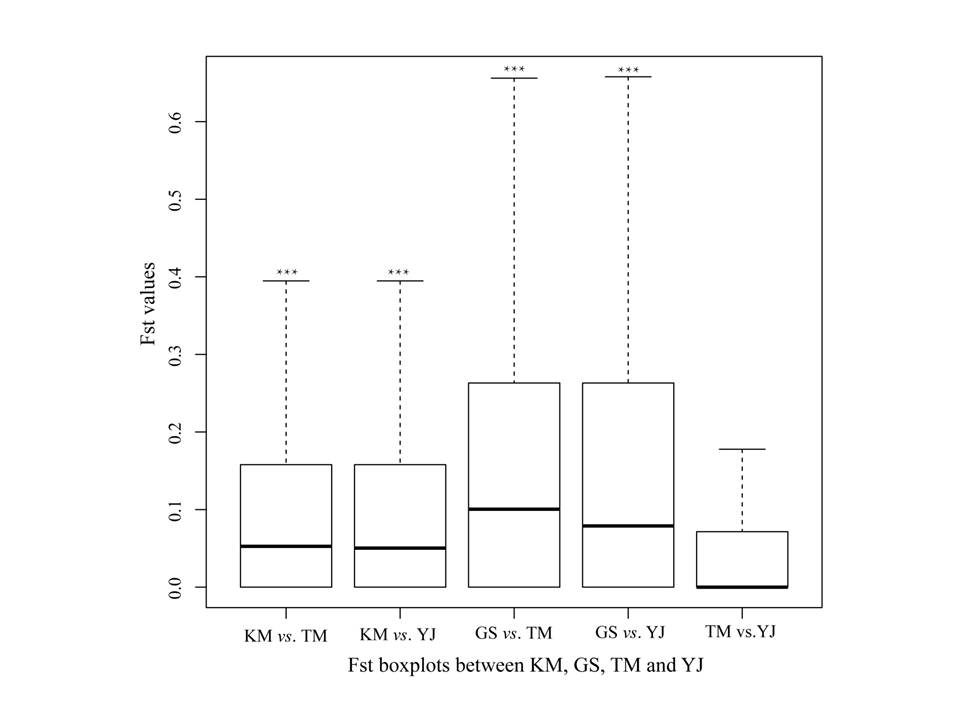


**Supplementary Fig. S4. Boxplots of the Fst calculated from different dog population pairs from Gou *et al.* (2014).** Based on the whole autosomal SNPs dataset of GS, KM, TM and YJ populations, we calculated Fst for each SNP and assessed the divergence levels (all P value were lower than 2.2E-16) between the values of Fst generated from each population pairs. Asterisk above whiskers means the significant levels of the divergence between the TM-YJ pair and another population pairs.


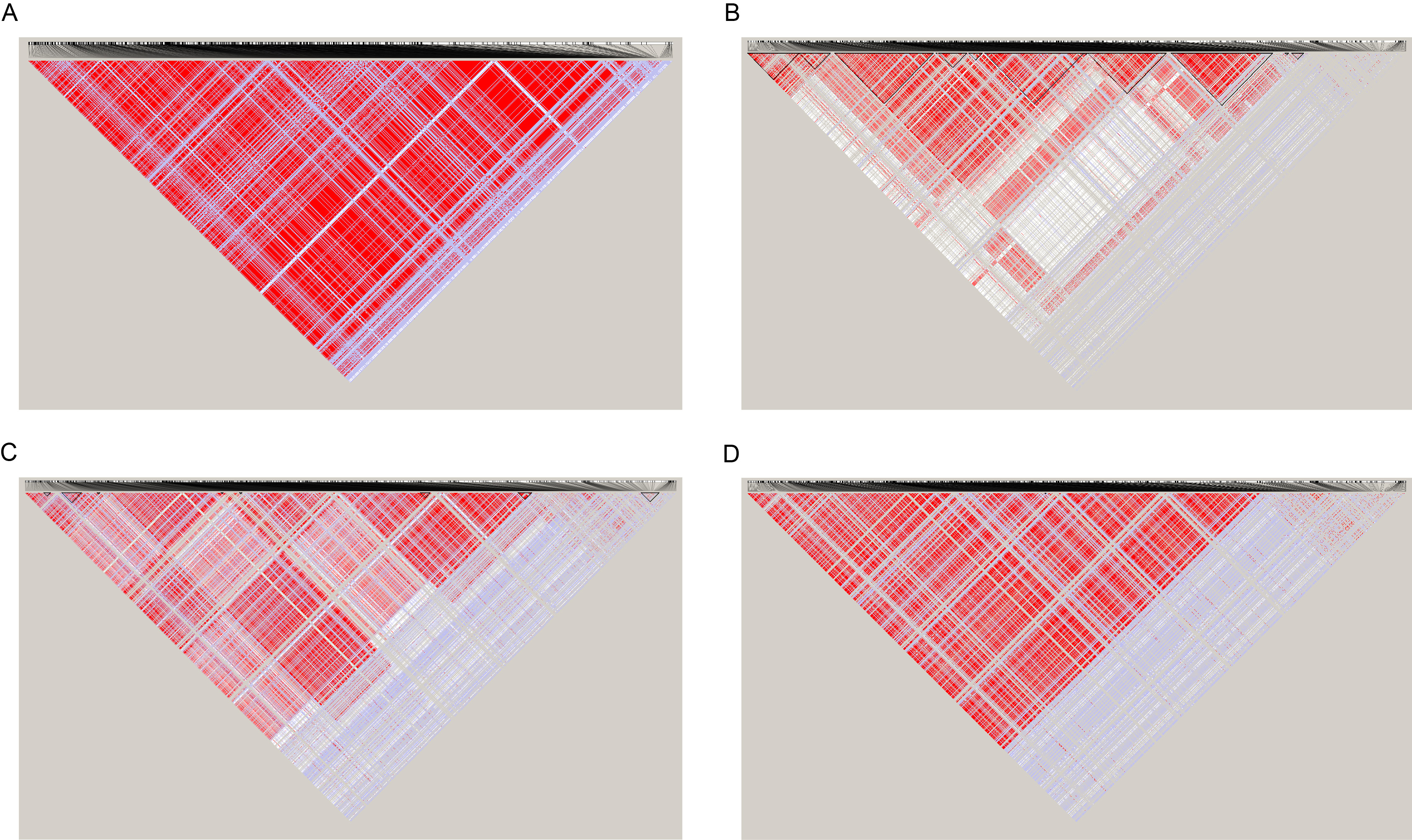


**Supplementary Fig. S5. Linkage Disequilibrium (LD) analysis around *AMOT* (~500k bp) for the four dog populations, TM, DQ, LJ and YJ from Gou *et al.* (2014).** Regions with “red colour” and “gray colour” represent strong and weak LD block, respectively. (A). LD analysis for TM population; (B). LD analysis for DQ population; (C). LD analysis for LJ population; (D). LD analysis for YJ population.


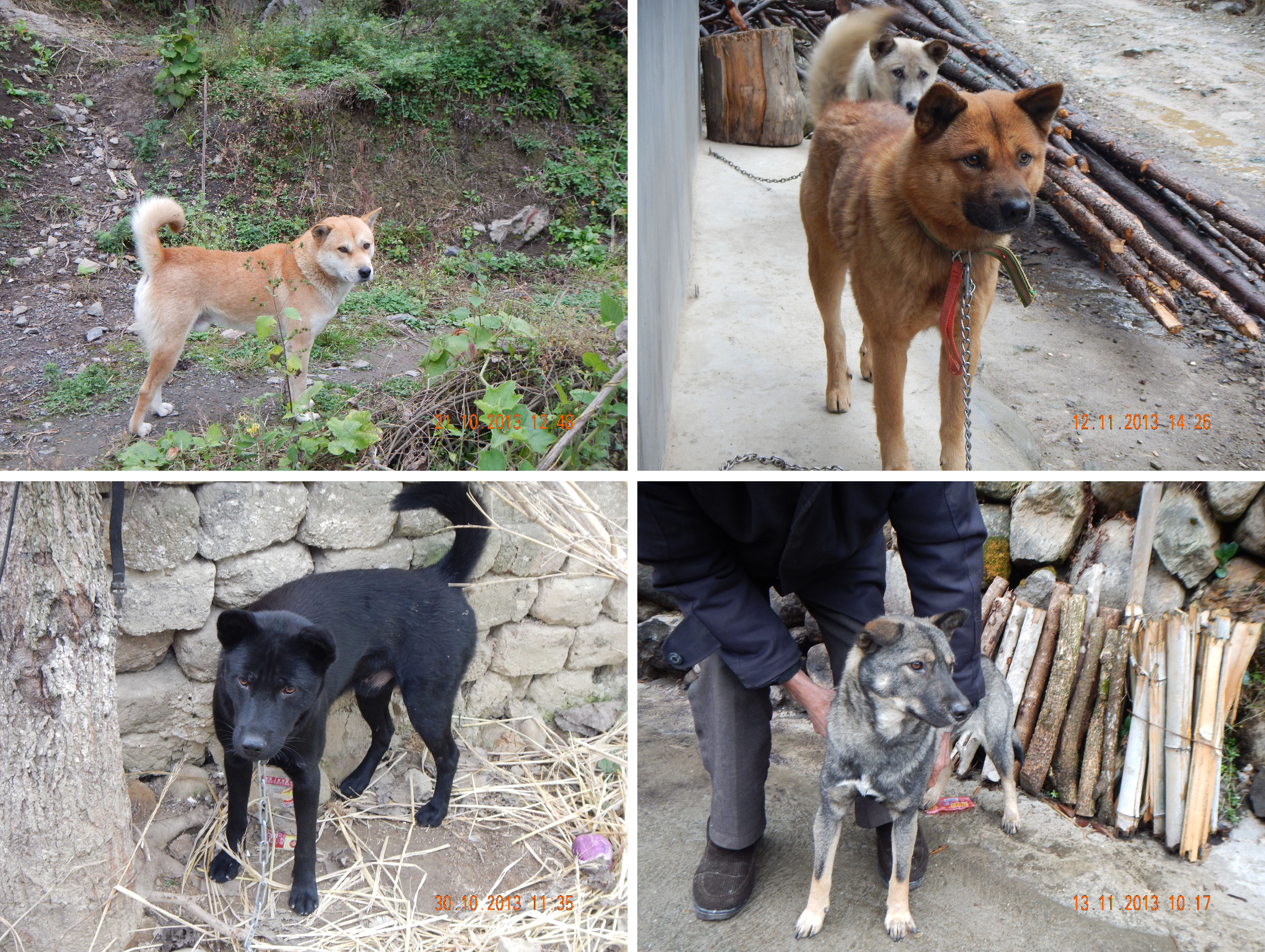


**Supplementary Fig. S6. Sample photos of our additionally collected dogs.** To avoid the genetic mixture with some modern breeds, our sampling work was carried out in remote villages. The collecting distance interval was 15km at minimum between each village.


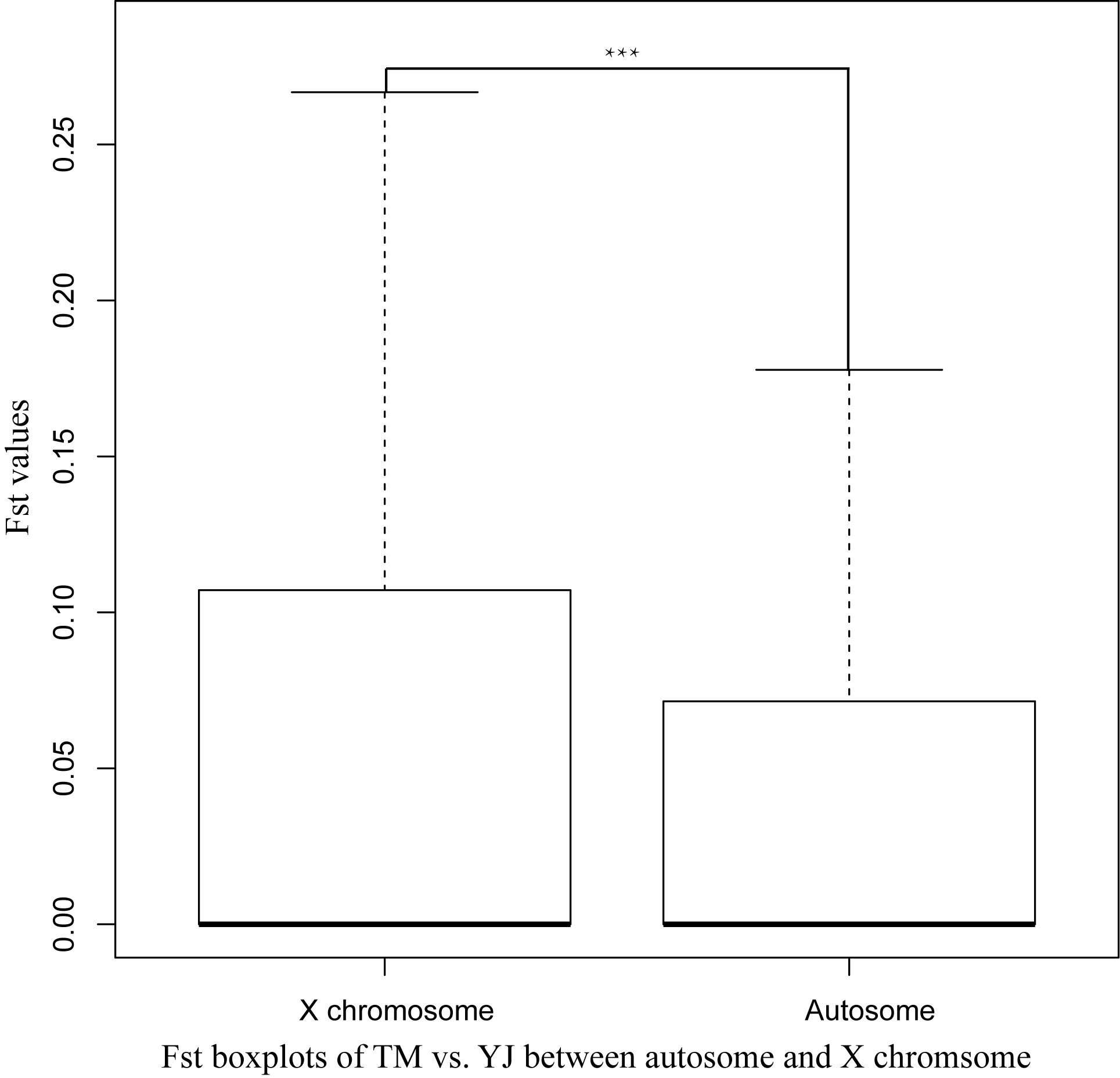


**Supplementary Fig. S7. Boxplots of the Fst values calculated from autosomal and allosomal SNPs for TM vs. YJ populations from Gou *et al.* (2014).** The significant level of the Fst divergence between autosome and X chromosome is P=2.2E-16.

**Supplementary Table S1. Sample information of the six dog populations from Gou *et al.* (2014)**

| **Population (abbreviation)** | **Location** | **Sample size** | **Altitude** |
| --- | --- | --- | --- |
| indigenous dog (YJ) | Yingjiang, Yunnan, China | 10 | 800 m |
| Kunming dog (KM) | Kunming, Yunnan, China | 10 | 1800 m |
| German Shephed (GS) | Kunming, Yunnan, China | 10 | 1800 m |
| indigenous dog (LJ-1) | Lijiang, Yunnan, China | 10 | 2400 m |
| indigenous dog (DQ) | Diqing, Yunnan, China | 10 | 3300 m |
| Tibetan Mastiff (TM) | Tibet, Qinghai, Yunnan, in China | 10 | 4380 m |

**Supplementary Table S2. Gender information of the six dog populations from Gou *et al.* (2014)**

| **Location** | **Population** | **Sample ID** | **Gender (F/M)** | **Altitude (m)** |
| --- | --- | --- | --- | --- |
| Yingjiang, Yunnan, China | YJ | YJ1 | M | 800 |
| Yingjiang, Yunnan, China | YJ | YJ2 | M | 800 |
| Yingjiang, Yunnan, China | YJ | YJ3 | F | 800 |
| Yingjiang, Yunnan, China | YJ | YJ4 | M | 800 |
| Yingjiang, Yunnan, China | YJ | YJ5 | F | 800 |
| Yingjiang, Yunnan, China | YJ | YJ6 | F | 800 |
| Yingjiang, Yunnan, China | YJ | YJ7 | M | 800 |
| Yingjiang, Yunnan, China | YJ | YJ8 | M | 800 |
| Yingjiang, Yunnan, China | YJ | YJ9 | F | 800 |
| Yingjiang, Yunnan, China | YJ | YJ10 | F | 800 |
| Kunming, Yunnan, China | KM | KM1 | F | 1800 |
| Kunming, Yunnan, China | KM | KM2 | F | 1800 |
| Kunming, Yunnan, China | KM | KM3 | F | 1800 |
| Kunming, Yunnan, China | KM | KM4 | F | 1800 |
| Kunming, Yunnan, China | KM | KM5 | F | 1800 |
| Kunming, Yunnan, China | KM | KM6 | M | 1800 |
| Kunming, Yunnan, China | KM | KM7 | M | 1800 |
| Kunming, Yunnan, China | KM | KM8 | M | 1800 |
| Kunming, Yunnan, China | KM | KM9 | M | 1800 |
| Kunming, Yunnan, China | KM | KM10 | M | 1800 |
| Kunming, Yunnan, China | GS | GS1 | F | 1800 |
| Kunming, Yunnan, China | GS | GS2 | F | 1800 |
| Kunming, Yunnan, China | GS | GS3 | F | 1800 |
| Kunming, Yunnan, China | GS | GS4 | F | 1800 |
| Kunming, Yunnan, China | GS | GS5 | F | 1800 |
| Kunming, Yunnan, China | GS | GS6 | M | 1800 |
| Kunming, Yunnan, China | GS | GS7 | M | 1800 |
| Kunming, Yunnan, China | GS | GS8 | M | 1800 |
| Kunming, Yunnan, China | GS | GS9 | M | 1800 |
| Kunming, Yunnan, China | GS | GS10 | M | 1800 |
| Lijiang, Yunnan, China | LJ-1 | LJ1-1 | M | 2400 |
| Lijiang, Yunnan, China | LJ-1 | LJ1-2 | M | 2400 |
| Lijiang, Yunnan, China | LJ-1 | LJ1-3 | F | 2400 |
| Lijiang, Yunnan, China | LJ-1 | LJ1-4 | F | 2400 |
| Lijiang, Yunnan, China | LJ-1 | LJ1-5 | F | 2400 |
| Lijiang, Yunnan, China | LJ-1 | LJ1-6 | F | 2400 |
| Lijiang, Yunnan, China | LJ-1 | LJ1-7 | F | 2400 |
| Lijiang, Yunnan, China | LJ-1 | LJ1-8 | M | 2400 |
| Lijiang, Yunnan, China | LJ-1 | LJ1-9 | M | 2400 |
| Lijiang, Yunnan, China | LJ-1 | LJ1-10 | M | 2400 |
| Diqing, Yunnan, China | DQ | DQ1 | F | 3300 |
| Diqing, Yunnan, China | DQ | DQ2 | F | 3300 |
| Diqing, Yunnan, China | DQ | DQ3 | M | 3300 |
| Diqing, Yunnan, China | DQ | DQ4 | M | 3300 |
| Diqing, Yunnan, China | DQ | DQ5 | F | 3300 |
| Diqing, Yunnan, China | DQ | DQ6 | F | 3300 |
| Diqing, Yunnan, China | DQ | DQ7 | M | 3300 |
| Diqing, Yunnan, China | DQ | DQ8 | M | 3300 |
| Diqing, Yunnan, China | DQ | DQ9 | F | 3300 |
| Diqing, Yunnan, China | DQ | DQ10 | M | 3300 |
| Tibet, Qinghai, Yunnan, in China | TM | TM1 | M | 4380 |
| Tibet, Qinghai, Yunnan, in China | TM | TM2 | F | 4380 |
| Tibet, Qinghai, Yunnan, in China | TM | TM3 | F | 4380 |
| Tibet, Qinghai, Yunnan, in China | TM | TM4 | M | 4380 |
| Tibet, Qinghai, Yunnan, in China | TM | TM5 | M | 4380 |
| Tibet, Qinghai, Yunnan, in China | TM | TM6 | M | 4380 |
| Tibet, Qinghai, Yunnan, in China | TM | TM7 | F | 4380 |
| Tibet, Qinghai, Yunnan, in China | TM | TM8 | M | 4380 |
| Tibet, Qinghai, Yunnan, in China | TM | TM9 | F | 4380 |
| Tibet, Qinghai, Yunnan, in China | TM | TM10 | F | 4380 |

**Supplementary Table S3. Summaries of the mapping results of the downloaded data sets from Gou *et al.* (2014).**

| **Sample^a^** | **Reads** | **Bases** | **Mapped reads** | **Mapped bases** | **Autosome**  **coverage^b^** | **X chromosome coverage^b^** |
| --- | --- | --- | --- | --- | --- | --- |
| GS1 | 3.82E+08 | 3.82E+10 | 3.81E+08 | 3.81E+10 | 14.22 | 14.27 |
| GS2 | 4.00E+08 | 4.00E+10 | 3.99E+08 | 3.99E+10 | 14.99 | 8.16 |
| GS3 | 3.91E+08 | 3.91E+10 | 3.90E+08 | 3.90E+10 | 14.63 | 14.77 |
| GS4 | 3.89E+08 | 3.89E+10 | 3.88E+08 | 3.88E+10 | 14.42 | 14.62 |
| GS5 | 3.75E+08 | 3.75E+10 | 3.74E+08 | 3.74E+10 | 13.82 | 14.07 |
| GS6 | 3.96E+08 | 3.96E+10 | 3.95E+08 | 3.95E+10 | 14.60 | 8.02 |
| GS7 | 4.12E+08 | 4.12E+10 | 4.11E+08 | 4.11E+10 | 15.29 | 8.44 |
| GS8 | 3.79E+08 | 3.79E+10 | 3.78E+08 | 3.78E+10 | 13.45 | 7.40 |
| GS9 | 4.14E+08 | 4.14E+10 | 4.13E+08 | 4.13E+10 | 15.34 | 8.45 |
| GS10 | 3.89E+08 | 3.89E+10 | 3.88E+08 | 3.88E+10 | 14.29 | 7.84 |
| KM1 | 2.92E+08 | 2.93E+10 | 2.90E+08 | 2.92E+10 | 11.01 | 10.95 |
| KM2 | 4.18E+08 | 4.22E+10 | 4.17E+08 | 4.21E+10 | 15.86 | 15.96 |
| KM3 | 4.01E+08 | 4.01E+10 | 4.00E+08 | 4.00E+10 | 15.02 | 15.18 |
| KM4 | 4.11E+08 | 4.11E+10 | 4.11E+08 | 4.11E+10 | 15.45 | 15.60 |
| KM5 | 4.08E+08 | 4.08E+10 | 4.07E+08 | 4.07E+10 | 15.24 | 15.43 |
| KM6 | 3.92E+08 | 3.96E+10 | 3.92E+08 | 3.95E+10 | 14.91 | 8.08 |
| KM7 | 4.39E+08 | 4.43E+10 | 4.38E+08 | 4.42E+10 | 16.50 | 8.89 |
| KM8 | 3.49E+08 | 3.52E+10 | 3.48E+08 | 3.51E+10 | 13.26 | 7.24 |
| KM9 | 3.56E+08 | 3.58E+10 | 3.55E+08 | 3.57E+10 | 13.56 | 7.31 |
| KM10 | 4.17E+08 | 4.22E+10 | 4.17E+08 | 4.21E+10 | 15.71 | 8.68 |
| YJ1 | 4.10E+08 | 4.10E+10 | 4.09E+08 | 4.09E+10 | 15.27 | 8.32 |
| YJ2 | 3.66E+08 | 3.66E+10 | 3.65E+08 | 3.65E+10 | 13.26 | 7.16 |
| YJ3 | 3.58E+08 | 3.58E+10 | 3.58E+08 | 3.58E+10 | 12.89 | 13.07 |
| YJ4 | 3.64E+08 | 3.64E+10 | 3.63E+08 | 3.63E+10 | 13.02 | 7.07 |
| YJ5 | 3.51E+08 | 3.51E+10 | 3.50E+08 | 3.50E+10 | 12.62 | 12.64 |
| YJ6 | 3.82E+08 | 3.82E+10 | 3.73E+08 | 3.73E+10 | 13.60 | 13.78 |
| YJ7 | 3.70E+08 | 3.70E+10 | 3.69E+08 | 3.69E+10 | 13.07 | 7.18 |
| YJ8 | 3.79E+08 | 3.79E+10 | 3.78E+08 | 3.78E+10 | 13.62 | 7.49 |
| YJ9 | 3.79E+08 | 3.79E+10 | 3.78E+08 | 3.78E+10 | 13.86 | 14.06 |
| YJ10 | 3.96E+08 | 3.96E+10 | 3.95E+08 | 3.95E+10 | 14.63 | 14.75 |
| LJ1 | 4.07E+08 | 4.07E+10 | 4.07E+08 | 4.07E+10 | 15.38 | 8.53 |
| LJ2 | 4.07E+08 | 4.07E+10 | 4.06E+08 | 4.06E+10 | 15.34 | 8.46 |
| LJ3 | 3.88E+08 | 3.88E+10 | 3.88E+08 | 3.88E+10 | 14.67 | 15.03 |
| LJ4 | 3.49E+08 | 3.49E+10 | 3.48E+08 | 3.48E+10 | 13.23 | 13.31 |
| LJ5 | 3.72E+08 | 3.72E+10 | 3.71E+08 | 3.71E+10 | 13.87 | 14.38 |
| LJ6 | 3.85E+08 | 3.85E+10 | 3.84E+08 | 3.84E+10 | 14.25 | 14.33 |
| LJ7 | 3.29E+08 | 3.29E+10 | 3.28E+08 | 3.28E+10 | 12.58 | 12.51 |
| LJ8 | 3.91E+08 | 3.91E+10 | 3.90E+08 | 3.90E+10 | 14.60 | 7.94 |
| LJ9 | 3.91E+08 | 3.91E+10 | 3.90E+08 | 3.90E+10 | 14.62 | 8.09 |
| LJ10 | 3.75E+08 | 3.75E+10 | 3.74E+08 | 3.74E+10 | 14.20 | 7.94 |
| DQ1 | 4.06E+08 | 4.06E+10 | 4.05E+08 | 4.05E+10 | 15.14 | 15.48 |
| DQ2 | 4.22E+08 | 4.22E+10 | 4.21E+08 | 4.21E+10 | 15.77 | 16.04 |
| DQ3 | 3.70E+08 | 3.70E+10 | 3.69E+08 | 3.69E+10 | 13.64 | 13.99 |
| DQ4 | 3.85E+08 | 3.85E+10 | 3.84E+08 | 3.84E+10 | 14.13 | 14.34 |
| DQ5 | 4.03E+08 | 4.03E+10 | 4.02E+08 | 4.02E+10 | 15.13 | 15.38 |
| DQ6 | 3.38E+08 | 3.38E+10 | 3.37E+08 | 3.37E+10 | 12.64 | 12.89 |
| DQ7 | 4.15E+08 | 4.15E+10 | 4.14E+08 | 4.14E+10 | 15.28 | 8.36 |
| DQ8 | 3.24E+08 | 3.24E+10 | 3.23E+08 | 3.23E+10 | 11.95 | 6.59 |
| DQ9 | 3.61E+08 | 3.65E+10 | 3.61E+08 | 3.64E+10 | 13.65 | 13.80 |
| DQ10 | 4.00E+08 | 4.00E+10 | 3.99E+08 | 3.99E+10 | 14.87 | 8.23 |
| TM1 | 3.89E+08 | 3.93E+10 | 3.88E+08 | 3.92E+10 | 14.52 | 8.05 |
| TM2 | 3.92E+08 | 3.64E+10 | 3.91E+08 | 3.63E+10 | 13.10 | 13.36 |
| TM3 | 4.01E+08 | 3.73E+10 | 4.00E+08 | 3.72E+10 | 13.35 | 13.68 |
| TM4 | 3.40E+08 | 3.40E+10 | 3.39E+08 | 3.39E+10 | 12.49 | 12.88 |
| TM5 | 3.64E+08 | 3.64E+10 | 3.63E+08 | 3.63E+10 | 13.38 | 7.39 |
| TM6 | 3.79E+08 | 3.79E+10 | 3.78E+08 | 3.78E+10 | 13.85 | 7.61 |
| TM7 | 4.06E+08 | 4.06E+10 | 4.05E+08 | 4.05E+10 | 15.13 | 15.41 |
| TM8 | 3.60E+08 | 3.60E+10 | 3.59E+08 | 3.59E+10 | 13.20 | 13.60 |
| TM9 | 4.07E+08 | 4.07E+10 | 4.06E+08 | 4.06E+10 | 15.15 | 15.43 |
| TM10 | 4.07E+08 | 4.07E+10 | 4.06E+08 | 4.06E+10 | 15.14 | 15.30 |

a. More details of samples information can be found in Guo *et al.* 2014;

b. Coverage was calculated after filtering out PCR duplicated reads, and bases with sequencing quality as well as mapping quality less than 20.

**Supplementary Table S4. Summaries of autosomal SNPs for the six populations from Gou *et al.* (2014)**

| **Populations** | **Total SNPs** | **Transition (Ts)** | **Transversion (Tv)** | **Ts/Tv Ratio** |
| --- | --- | --- | --- | --- |
| **TM** | 10,001,578 | 6,839,286 | 3,162,292 | 2.16 |
| **DQ** | 9,943,006 | 6,798,948 | 3,144,058 | 2.16 |
| **LJ** | 10,443,849 | 7,132,,912 | 3,310,937 | 2.15 |
| **KM** | 8,128,809 | 5,548,434 | 2,580,375 | 2.15 |
| **GS** | 5,771,915 | 3,918,656 | 1,853,259 | 2.11 |
| **YJ** | 10,399,210 | 7,106,041 | 3,293,169 | 2.16 |
| **All populations** | 14,303,817 | 9,768,736 | 4,535,081 | 2.15 |

**Supplementary Table S5. Summaries of X chromosome SNPs for the six populations from Gou *et al.* (2014)**

| **Populations** | **Total SNPs** | **Transition(Ts)** | **Tranversion(Tv)** | **Ts/Tv Ratio** |
| --- | --- | --- | --- | --- |
| **TM** | 293,233 | 188,390 | 104,843 | 1.80 |
| **DQ** | 294,116 | 189,276 | 104,840 | 1.81 |
| **LJ** | 318,848 | 204,055 | 114,793 | 1.78 |
| **KM** | 241,567 | 153,766 | 87,801 | 1.75 |
| **GS** | 191,051 | 120,461 | 70,590 | 1.71 |
| **YJ** | 314,562 | 201,254 | 113,308 | 1.78 |
| **All populations** | 435,757 | 278,050 | 157,707 | 1.76 |

**Supplementary Table S6. The annotations of X chromosome SNPs for the six populations from Gou *et al.* (2014)**

| **Population** | **Intergenic^a^** | **ncRNA^b^** | **UTR^c^** | **Intronic** | **Splicing** | **Exonic^d^** | | |
| --- | --- | --- | --- | --- | --- | --- | --- | --- |
|  |  |  |  |  |  | **Synonymous** | **Non-synonymous** | **Stop altering^e^** |
| **TM** | 224,563 | 341 | 1,277 | 63,257 | 11 | 2,494 | 1,280 | 10 |
| **DQ** | 223,728 | 333 | 1,320 | 64,882 | 11 | 2,532 | 1,299 | 11 |
| **LJ** | 243,551 | 386 | 1,365 | 69,642 | 13 | 2,546 | 1,332 | 13 |
| **KM** | 182,376 | 311 | 1,131 | 54,045 | 11 | 2,435 | 1,247 | 11 |
| **GS** | 143,090 | 251 | 876 | 43,412 | 11 | 2,305 | 1,100 | 6 |
| **YJ** | 239,657 | 373 | 1,302 | 69,306 | 13 | 2,552 | 1,345 | 14 |

^a^ Including "intergenic", "upstream" and "downstream" given by ANNOVAR.

^b^ Including "ncRNA_exonic", "ncRNA_intronic", "ncRNA_splicing" and "ncRNA_exonic;splicing"

^c^ Including "UTR5” and "UTR3".

^d^ Including "exonic" and "exonic;splicing".

^e^ Including "stopgain SNV" and "stoploss SNV".

**Supplementary Table S7. Summaries of the 24 outlier genes identified from TM vs. YJ populations from Gou *et al.* (2014)**

| **Gene IDs** | **Gene Symbols** | **Intervals of Genes** | **Number of SNPs with outlier *P*** | **Number of SNPs with outlier Fst** |
| --- | --- | --- | --- | --- |
| ENSCAFG00000013658 | *NR0B1* | 25396523-25401715 | 1 | 1 |
| ENSCAFG00000014251 | *DDX3X* | 35759500-35772776 | 1 | 1 |
| ENSCAFG00000015146 | *SYN1* | 41188745-41241624 | 4 | 4 |
| ENSCAFG00000016028 | *SHROOM4* | 43370321-43639302 | 4 | 1 |
| ENSCAFG00000016720 | *STARD8* | 53128302-53211164 | 2 | 2 |
| ENSCAFG00000017252 | *ATRX* | 59772067-60107186 | 1 | 1 |
| ENSCAFG00000017380 | *HDX* | 65206566-65429368 | 18 | 18 |
| ENSCAFG00000017479 | *DIAPH2* | 69994661-71044235 | 69 | 68 |
| ENSCAFG00000018083 | *TMEM164* | 83219513-83385054 | 2 | 2 |
| ENSCAFG00000018179 | *DCX* | 84408987-84517827 | 184 | 185 |
| ENSCAFG00000018196 | *ALG13* | 84773778-84825410 | 118 | 115 |
| ENSCAFG00000018204 | *TRPC5* | 84842015-84989591 | 350 | 335 |
| ENSCAFG00000018212 | *ZCCHC16* | 85512467-85513375 | 3 | 3 |
| ENSCAFG00000018215 | *LHFPL1* | 85594587-85648714 | 135 | 128 |
| ENSCAFG00000018218 | *AMOT* | 85742216-85789759 | 87 | 86 |
| ENSCAFG00000018777 | *ENOX2* | 101742407-101914196 | 5 | 5 |
| ENSCAFG00000018785 | *ARHGAP36* | 102196308-102227615 | 3 | 3 |
| ENSCAFG00000018826 | *STK26* | 102961508-103026811 | 1 | 1 |
| ENSCAFG00000018829 | *FRMD7* | 103028814-103079022 | 1 | 1 |
| ENSCAFG00000018841 | *MBNL3* | 103266497-103326805 | 1 | 1 |
| ENSCAFG00000018864 | *GPC3* | 104313240-104751483 | 3 | 3 |
| ENSCAFG00000019245 | *BCAP31* | 121515152-121587576 | 1 | 1 |
| ENSCAFG00000024515 | *ARSH* | 1567027-1587983 | 2 | 3 |
| ENSCAFG00000024670 | *ASMT* | 1012952-1025263 | 5 | 5 |

**Supplementary Table S8. Summaries of the 64 outlier genes identified from TM&DQ vs. YJ populations from Gou *et al.*** (2014)

| **Gene IDs** | **Gene Symbols** | **Intervals of Genes** | **Number of SNPs  with outlier P** | **Number of SNPs  with outlier Fst** |
| --- | --- | --- | --- | --- |
| ENSCAFG00000011041 | *CSF2RA* | 832167-857143 | 2 | 8 |
| ENSCAFG00000011278 | *HDHD1* | 4248224-4290551 | 2 | 6 |
| ENSCAFG00000011287 | *STS* | 4363261-4466619 | 1 | 1 |
| ENSCAFG00000011595 | *ARHGAP6* | 7704809-7952234 | 1 | 2 |
| ENSCAFG00000011627 | *FRMPD4* | 9031850-9229271 | 4 | 4 |
| ENSCAFG00000012774 | *REPS2* | 13089118-13307090 | 3 | 3 |
| ENSCAFG00000013840 | *CXORF59* | 31044315-31104634 | 5 | 5 |
| ENSCAFG00000014023 | *OTC* | 33130884-33202534 | 1 | 5 |
| ENSCAFG00000014251 | *DDX3X* | 35759500-35772776 | 1 | 1 |
| ENSCAFG00000014469 | *EFHC2* | 38133229-38297269 | 1 | 1 |
| ENSCAFG00000015146 | *SYN1* | 41188745-41241624 | 1 | 1 |
| ENSCAFG00000015170 | *ZNF81* | 41353292-41457339 | 3 | 4 |
| ENSCAFG00000016028 | *SHROOM4* | 43370321-43639302 | 1 | 1 |
| ENSCAFG00000016064 | *MAGED1* | 44395132-44458891 | 4 | 4 |
| ENSCAFG00000016709 | *YIPF6* | 53025927-53045227 | 1 | 1 |
| ENSCAFG00000016746 | *FAM155B* | 53969605-53998323 | 1 | 1 |
| ENSCAFG00000016752 | *AWAT2* | 54516776-54531450 | 1 | 1 |
| ENSCAFG00000016894 | *TEX11* | 54958579-55252726 | 2 | 2 |
| ENSCAFG00000016921 | *SNX12* | 55267765-55450938 | 2 | 2 |
| ENSCAFG00000017252 | *ATRX* | 59772067-60107186 | 18 | 2 |
| ENSCAFG00000017263 | *ATP7A* | 60203319-60356690 | 1 | 1 |
| ENSCAFG00000017373 | *RPS6KA6* | 64921230-65111098 | 14 | 17 |
| ENSCAFG00000017380 | *HDX* | 65206566-65429368 | 31 | 31 |
| ENSCAFG00000017401 | *POF1B* | 66233161-66342044 | 1 | 1 |
| ENSCAFG00000017419 | *DACH2* | 67256314-67698597 | 14 | 2 |
| ENSCAFG00000017445 | *KLHL4* | 67949053-68110330 | 1 | 1 |
| ENSCAFG00000017479 | *DIAPH2* | 69994661-71044235 | 247 | 253 |
| ENSCAFG00000018009 | *ATG4A* | 81668224-81687622 | 1 | 1 |
| ENSCAFG00000018020 | *COL4A5* | 81982070-82250078 | 16 | 16 |
| ENSCAFG00000018083 | *TMEM164* | 83219513-83385054 | 59 | 59 |
| ENSCAFG00000018179 | *DCX* | 84408987-84517827 | 139 | 140 |
| ENSCAFG00000018196 | *ALG13* | 84773778-84825410 | 16 | 16 |
| ENSCAFG00000018204 | *TRPC5* | 84842015-84989591 | 18 | 18 |
| ENSCAFG00000018215 | *LHFPL1* | 85594587-85648714 | 18 | 18 |
| ENSCAFG00000018218 | *AMOT* | 85742216-85789759 | 1 | 1 |
| ENSCAFG00000018378 | *SLC25A43* | 91471708-91509533 | 2 | 2 |
| ENSCAFG00000018673 | *SMARCA1* | 100677888-100749065 | 3 | 3 |
| ENSCAFG00000018700 | *XPNPEP2* | 100951888-100978445 | 8 | 8 |
| ENSCAFG00000018735 | *ELF4* | 101218419-101259567 | 16 | 16 |
| ENSCAFG00000018759 | *ZNF280C* | 101338204-101404318 | 2 | 1 |
| ENSCAFG00000018777 | *ENOX2* | 101742407-101914196 | 5 | 5 |
| ENSCAFG00000018785 | *ARHGAP36* | 102196308-102227615 | 3 | 3 |
| ENSCAFG00000018799 | *IGSF1* | 102359983-102373889 | 1 | 1 |
| ENSCAFG00000018829 | *FRMD7* | 103028814-103079022 | 1 | 1 |
| ENSCAFG00000018841 | *MBNL3* | 103266497-103326805 | 1 | 1 |
| ENSCAFG00000018864 | *GPC3* | 104313240-104751483 | 7 | 3 |
| ENSCAFG00000018952 | *ARHGEF6* | 107029681-107133082 | 5 | 6 |
| ENSCAFG00000019103 | *TMEM185A* | 117951803-117966408 | 1 | 1 |
| ENSCAFG00000019230 | *ATP2B3* | 121361391-121402609 | 1 | 3 |
| ENSCAFG00000019245 | *BCAP31* | 121515152-121587576 | 1 | 1 |
| ENSCAFG00000023562 | *DMD* | 26290714-28333431 | 11 | 4 |
| ENSCAFG00000023600 | *CXorf30* | 31252878-31391015 | 1 | 4 |
| ENSCAFG00000024088 | *CXorf22* | 30880536-30946981 | 38 | 44 |
| ENSCAFG00000024515 | *ARSH* | 1567027-1587983 | 1 | 2 |
| ENSCAFG00000024670 | *ASMT* | 1012952-1025263 | 15 | 28 |
| ENSCAFG00000030544 | *PJA1* | 53606560-53614345 | 1 | 1 |
| ENSCAFG00000031215 | *CXorf36* | 38994591-39039315 | 2 | 2 |
| ENSCAFG00000027478 | *U6* | 91403440-91403543 | 1 | 1 |
| ENSCAFG00000029984 | *ZFP92* | 121237884-121248929 | 3 | 3 |
| ENSCAFG00000018826 | *STK26* | 102961508-103026811 | 1 | 1 |
| ENSCAFG00000011251 | *NLGN4X* | 3412434-3676064 | 13 | 33 |
| ENSCAFG00000014051 | *TSPAN7* | 33336418-33465096 | 1 | 1 |
| ENSCAFG00000017103 | *TAF1* | 55693086-55765038 | 1 | 1 |
| ENSCAFG00000016831 | *KIF4A* | 54739573-54863225 | 2 | 2 |

**Supplementary Table S9. Sample information of our collected 11 dog populations.**

| **Population (abbreviation)** | **Location** | **Sample size** | **Altitude** |
| --- | --- | --- | --- |
| indigenous dog (HEB) | Harbin, Heilongjiang, China | 17 | 300 m |
| indigenous dog (PZ) | Pengze, Jiangxi, China | 14 | 300 m |
| indigenous dog (YA) | Ya an, Sichuan, China | 25 | 600 m |
| indigenous dog (CD) | Chengde, Hebei, China | 22 | 1000 m |
| indigenous dog (XA) | Xi an, Shanxi, China | 18 | 1000 m |
| indigenous dog (SM) | Simao, Yunnan, China | 19 | 1300 m |
| indigenous dog (YX) | Yuxi, Yunnan, China | 10 | 1600 m |
| indigenous dog (LJ-2) | Lijiang, Yunnan, China | 11 | 2500 m |
| Tibetan Mastiff (MQ) | Maqu, Gansu, China | 10 | 3600 m |
| Tibetan dog (HY) | Hongyuan, Sichuan, China | 11 | 3700 m |
| Tibetan Mastiff (YS) | Yushu, Qinghai, China | 18 | 4000 m |

**Supplementary Table S10. Gender information of our collected 11 dog populations**

| **Location** | **Population** | **Sample ID** | **Gender (F/M)** | **Altitude (m)** |
| --- | --- | --- | --- | --- |
| Harbin, Heilongjiang, China | HEB | HEB1 | F | 300 |
| Harbin, Heilongjiang, China | HEB | HEB2 | F | 300 |
| Harbin, Heilongjiang, China | HEB | HEB3 | F | 300 |
| Harbin, Heilongjiang, China | HEB | HEB4 | M | 300 |
| Harbin, Heilongjiang, China | HEB | HEB5 | M | 300 |
| Harbin, Heilongjiang, China | HEB | HEB6 | M | 300 |
| Harbin, Heilongjiang, China | HEB | HEB7 | F | 300 |
| Harbin, Heilongjiang, China | HEB | HEB8 | F | 300 |
| Harbin, Heilongjiang, China | HEB | HEB9 | F | 300 |
| Harbin, Heilongjiang, China | HEB | HEB10 | M | 300 |
| Harbin, Heilongjiang, China | HEB | HEB11 | F | 300 |
| Harbin, Heilongjiang, China | HEB | HEB12 | M | 300 |
| Harbin, Heilongjiang, China | HEB | HEB13 | M | 300 |
| Harbin, Heilongjiang, China | HEB | HEB14 | M | 300 |
| Harbin, Heilongjiang, China | HEB | HEB15 | F | 300 |
| Harbin, Heilongjiang, China | HEB | HEB16 | F | 300 |
| Harbin, Heilongjiang, China | HEB | HEB17 | M | 300 |
| Pengze, Jiangxi, China | PZ | PZ1 | M | 300 |
| Pengze, Jiangxi, China | PZ | PZ2 | F | 300 |
| Pengze, Jiangxi, China | PZ | PZ3 | M | 300 |
| Pengze, Jiangxi, China | PZ | PZ4 | M | 300 |
| Pengze, Jiangxi, China | PZ | PZ5 | M | 300 |
| Pengze, Jiangxi, China | PZ | PZ6 | F | 300 |
| Pengze, Jiangxi, China | PZ | PZ7 | F | 300 |
| Pengze, Jiangxi, China | PZ | PZ8 | M | 300 |
| Pengze, Jiangxi, China | PZ | PZ9 | F | 300 |
| Pengze, Jiangxi, China | PZ | PZ10 | F | 300 |
| Pengze, Jiangxi, China | PZ | PZ11 | F | 300 |
| Pengze, Jiangxi, China | PZ | PZ12 | M | 300 |
| Pengze, Jiangxi, China | PZ | PZ13 | M | 300 |
| Pengze, Jiangxi, China | PZ | PZ14 | F | 300 |
| Ya an, Sichuan, China | YA | YA1 | M | 600 |
| Ya an, Sichuan, China | YA | YA2 | M | 600 |
| Ya an, Sichuan, China | YA | YA3 | M | 600 |
| Ya an, Sichuan, China | YA | YA4 | M | 600 |
| Ya an, Sichuan, China | YA | YA5 | M | 600 |
| Ya an, Sichuan, China | YA | YA6 | M | 600 |
| Ya an, Sichuan, China | YA | YA7 | M | 600 |
| Ya an, Sichuan, China | YA | YA8 | M | 600 |
| Ya an, Sichuan, China | YA | YA9 | M | 600 |
| Ya an, Sichuan, China | YA | YA10 | F | 600 |
| Ya an, Sichuan, China | YA | YA11 | M | 600 |
| Ya an, Sichuan, China | YA | YA12 | M | 600 |
| Ya an, Sichuan, China | YA | YA13 | M | 600 |
| Ya an, Sichuan, China | YA | YA14 | M | 600 |
| Ya an, Sichuan, China | YA | YA15 | F | 600 |
| Ya an, Sichuan, China | YA | YA16 | M | 600 |
| Ya an, Sichuan, China | YA | YA17 | M | 600 |
| Ya an, Sichuan, China | YA | YA18 | M | 600 |
| Ya an, Sichuan, China | YA | YA19 | M | 600 |
| Ya an, Sichuan, China | YA | YA20 | M | 600 |
| Ya an, Sichuan, China | YA | YA21 | M | 600 |
| Ya an, Sichuan, China | YA | YA22 | M | 600 |
| Ya an, Sichuan, China | YA | YA23 | M | 600 |
| Ya an, Sichuan, China | YA | YA24 | M | 600 |
| Ya an, Sichuan, China | YA | YA25 | M | 600 |
| Chengde, Hebei, China | CD | CD1 | F | 1000 |
| Chengde, Hebei, China | CD | CD2 | F | 1000 |
| Chengde, Hebei, China | CD | CD3 | F | 1000 |
| Chengde, Hebei, China | CD | CD4 | F | 1000 |
| Chengde, Hebei, China | CD | CD5 | F | 1000 |
| Chengde, Hebei, China | CD | CD6 | F | 1000 |
| Chengde, Hebei, China | CD | CD7 | F | 1000 |
| Chengde, Hebei, China | CD | CD8 | F | 1000 |
| Chengde, Hebei, China | CD | CD9 | F | 1000 |
| Chengde, Hebei, China | CD | CD10 | F | 1000 |
| Chengde, Hebei, China | CD | CD11 | F | 1000 |
| Chengde, Hebei, China | CD | CD12 | F | 1000 |
| Chengde, Hebei, China | CD | CD13 | M | 1000 |
| Chengde, Hebei, China | CD | CD14 | M | 1000 |
| Chengde, Hebei, China | CD | CD15 | M | 1000 |
| Chengde, Hebei, China | CD | CD16 | M | 1000 |
| Chengde, Hebei, China | CD | CD17 | M | 1000 |
| Chengde, Hebei, China | CD | CD18 | M | 1000 |
| Chengde, Hebei, China | CD | CD19 | M | 1000 |
| Chengde, Hebei, China | CD | CD20 | M | 1000 |
| Chengde, Hebei, China | CD | CD21 | F | 1000 |
| Chengde, Hebei, China | CD | CD22 | F | 1000 |
| Xi an, Shanxi, China | XA | XA1 | F | 1000 |
| Xi an, Shanxi, China | XA | XA2 | M | 1000 |
| Xi an, Shanxi, China | XA | XA3 | F | 1000 |
| Xi an, Shanxi, China | XA | XA4 | F | 1000 |
| Xi an, Shanxi, China | XA | XA5 | F | 1000 |
| Xi an, Shanxi, China | XA | XA6 | F | 1000 |
| Xi an, Shanxi, China | XA | XA7 | M | 1000 |
| Xi an, Shanxi, China | XA | XA8 | F | 1000 |
| Xi an, Shanxi, China | XA | XA9 | M | 1000 |
| Xi an, Shanxi, China | XA | XA10 | M | 1000 |
| Xi an, Shanxi, China | XA | XA11 | M | 1000 |
| Xi an, Shanxi, China | XA | XA12 | F | 1000 |
| Xi an, Shanxi, China | XA | XA13 | M | 1000 |
| Xi an, Shanxi, China | XA | XA14 | M | 1000 |
| Xi an, Shanxi, China | XA | XA15 | M | 1000 |
| Xi an, Shanxi, China | XA | XA16 | M | 1000 |
| Xi an, Shanxi, China | XA | XA17 | M | 1000 |
| Xi an, Shanxi, China | XA | XA18 | F | 1000 |
| Simao, Yunnan, China | SM | SM1 | M | 1300 |
| Simao, Yunnan, China | SM | SM2 | F | 1300 |
| Simao, Yunnan, China | SM | SM3 | F | 1300 |
| Simao, Yunnan, China | SM | SM4 | F | 1300 |
| Simao, Yunnan, China | SM | SM5 | F | 1300 |
| Simao, Yunnan, China | SM | SM6 | F | 1300 |
| Simao, Yunnan, China | SM | SM7 | M | 1300 |
| Simao, Yunnan, China | SM | SM8 | M | 1300 |
| Simao, Yunnan, China | SM | SM9 | M | 1300 |
| Simao, Yunnan, China | SM | SM10 | M | 1300 |
| Simao, Yunnan, China | SM | SM11 | F | 1300 |
| Simao, Yunnan, China | SM | SM12 | F | 1300 |
| Simao, Yunnan, China | SM | SM13 | F | 1300 |
| Simao, Yunnan, China | SM | SM14 | F | 1300 |
| Simao, Yunnan, China | SM | SM15 | F | 1300 |
| Simao, Yunnan, China | SM | SM16 | F | 1300 |
| Simao, Yunnan, China | SM | SM17 | M | 1300 |
| Simao, Yunnan, China | SM | SM18 | M | 1300 |
| Simao, Yunnan, China | SM | SM19 | M | 1300 |
| Yuxi, Yunnan, China | YX | YX1 | M | 1600 |
| Yuxi, Yunnan, China | YX | YX2 | F | 1600 |
| Yuxi, Yunnan, China | YX | YX3 | F | 1600 |
| Yuxi, Yunnan, China | YX | YX4 | F | 1600 |
| Yuxi, Yunnan, China | YX | YX5 | M | 1600 |
| Yuxi, Yunnan, China | YX | YX6 | F | 1600 |
| Yuxi, Yunnan, China | YX | YX7 | F | 1600 |
| Yuxi, Yunnan, China | YX | YX8 | M | 1600 |
| Yuxi, Yunnan, China | YX | YX9 | F | 1600 |
| Yuxi, Yunnan, China | YX | YX10 | M | 1600 |
| Lijiang, Yunnan, China | LJ2 | LJ2_1 | F | 2500 |
| Lijiang, Yunnan, China | LJ2 | LJ2_2 | M | 2500 |
| Lijiang, Yunnan, China | LJ2 | LJ2_3 | F | 2500 |
| Lijiang, Yunnan, China | LJ2 | LJ2_4 | M | 2500 |
| Lijiang, Yunnan, China | LJ2 | LJ2_5 | M | 2500 |
| Lijiang, Yunnan, China | LJ2 | LJ2_6 | F | 2500 |
| Lijiang, Yunnan, China | LJ2 | LJ2_7 | M | 2500 |
| Lijiang, Yunnan, China | LJ2 | LJ2_8 | F | 2500 |
| Lijiang, Yunnan, China | LJ2 | LJ2_9 | M | 2500 |
| Lijiang, Yunnan, China | LJ2 | LJ2_10 | M | 2500 |
| Lijiang, Yunnan, China | LJ2 | LJ2_11 | F | 2500 |
| Maqu, Gansu, China | MQ | MQ1 | F | 3600 |
| Maqu, Gansu, China | MQ | MQ2 | F | 3600 |
| Maqu, Gansu, China | MQ | MQ3 | M | 3600 |
| Maqu, Gansu, China | MQ | MQ4 | F | 3600 |
| Maqu, Gansu, China | MQ | MQ5 | F | 3600 |
| Maqu, Gansu, China | MQ | MQ6 | M | 3600 |
| Maqu, Gansu, China | MQ | MQ7 | F | 3600 |
| Maqu, Gansu, China | MQ | MQ8 | F | 3600 |
| Maqu, Gansu, China | MQ | MQ9 | M | 3600 |
| Maqu, Gansu, China | MQ | MQ10 | F | 3600 |
| Hongyuan, Sichuan, China | HY | HY1 | F | 3700 |
| Hongyuan, Sichuan, China | HY | HY2 | F | 3700 |
| Hongyuan, Sichuan, China | HY | HY3 | F | 3700 |
| Hongyuan, Sichuan, China | HY | HY4 | M | 3700 |
| Hongyuan, Sichuan, China | HY | HY5 | M | 3700 |
| Hongyuan, Sichuan, China | HY | HY6 | F | 3700 |
| Hongyuan, Sichuan, China | HY | HY7 | F | 3700 |
| Hongyuan, Sichuan, China | HY | HY8 | M | 3700 |
| Hongyuan, Sichuan, China | HY | HY9 | F | 3700 |
| Hongyuan, Sichuan, China | HY | HY10 | F | 3700 |
| Hongyuan, Sichuan, China | HY | HY11 | F | 3700 |
| Yushu, Qinghai, China | YS | YS1 | F | 4000 |
| Yushu, Qinghai, China | YS | YS2 | F | 4000 |
| Yushu, Qinghai, China | YS | YS3 | F | 4000 |
| Yushu, Qinghai, China | YS | YS4 | M | 4000 |
| Yushu, Qinghai, China | YS | YS5 | M | 4000 |
| Yushu, Qinghai, China | YS | YS6 | M | 4000 |
| Yushu, Qinghai, China | YS | YS7 | M | 4000 |
| Yushu, Qinghai, China | YS | YS8 | F | 4000 |
| Yushu, Qinghai, China | YS | YS9 | F | 4000 |
| Yushu, Qinghai, China | YS | YS10 | F | 4000 |
| Yushu, Qinghai, China | YS | YS11 | F | 4000 |
| Yushu, Qinghai, China | YS | YS12 | F | 4000 |
| Yushu, Qinghai, China | YS | YS13 | M | 4000 |
| Yushu, Qinghai, China | YS | YS14 | M | 4000 |
| Yushu, Qinghai, China | YS | YS15 | M | 4000 |
| Yushu, Qinghai, China | YS | YS16 | M | 4000 |
| Yushu, Qinghai, China | YS | YS17 | M | 4000 |
| Yushu, Qinghai, China | YS | YS18 | F | 4000 |
